# *Branchiosyllis*, *Haplosyllis*, *Opisthosyllis *and *Trypanosyllis* (Annelida: Syllidae) from Brazil, with the Description of Two New Species

**DOI:** 10.1371/journal.pone.0153442

**Published:** 2016-05-04

**Authors:** Karla Paresque, Marcelo Veronesi Fukuda, João Miguel de Matos Nogueira

**Affiliations:** 1 Departamento de Zoologia, Instituto de Biociências, Universidade de São Paulo, São Paulo, São Paulo, Brazil; 2 Departamento de Zoologia, Instituto de Ciências Biológicas, Universidade Federal de Juiz de Fora, Juiz de Fora, Minas Gerais, Brazil; Institut Maurice Lamontagne, CANADA

## Abstract

Brazilian specimens of *Branchiosyllis* cf. *exilis*, *B*. *tamandarensis* **sp. n.**, *Haplosyllis lattigae* **sp. n.**, *H*. *loboi*, *Opisthosyllis brunnea* and *O*. *viridis* are described and illustrated herein, from recently collected material; also, the distributions of *Haplosyllis amphimedonicola* and *H*. *rosenalessoae* are expanded to other localities in the states of Paraíba and Pernambuco. *Branchiosyllis tamandarensis* **sp. n.** was found associated with sponges and is characterized by having a flattened, ribbon-like body, with longitudinal line of mid-dorsal papillae, peristomium dorsally inconspicuous, branchiae with up to six lobes, branchiae and ungulae on all parapodia, and falcigers absent. *Haplosyllis lattigae* **sp. n.** is characterized by having two kinds of chaetae with different sizes and shapes per parapodium, papillate dorsum from midbody, and midbody dorsal cirri alternating in length. Additionally, we provide keys to the Brazilian species of *Branchiosyllis*, *Haplosyllis* and *Opisthosyllis*, comparative tables of the new species described herein and selected similar congeners, and the first record for *Trypanosyllis zebra* in the states of Espírito Santo and Paraíba.

## Introduction

Syllidae Grube, 1850 is one of the most diverse families of polychaetes, with 74 valid genera and more than 700 species [1]. The family is represented by very abundant animals living epibiontically on various substrata, from hard to soft bottoms, and from intertidal to abyssal depths[1,2]. The subfamily Syllinae Grube, 1850, with about 260 species and 20 genera [1], is monophyletic [3]. According to the classification suggested by [3], the subfamily is characterized by reproducing schizogamically by scissiparity, with distinct types of stolons, corresponding to the internal monophyletic groups. Schyzogamy is also found in the Autolytinae Langerhans, 1879, but originating independently [3]. The results of [3] also suggests a close relationship between *Branchiosyllis* Ehlers, 1887 and *Haplosyllis* Langerhans, 1879, as both genera have species living in association with other organisms and have modified chaetae, which could be considered as a particular adaptation to symbiotic life styles [2,4–7]. In contraposition, *Opisthosyllis* Langerhans, 1879 and *Trypanosyllis* Claparède, 1864 are more closely related to the remaining genera of Syllinae. A historical approach and a general introduction to the subfamily is given by [6].

Up to the present, 8 genera and 47 species of the subfamily were recorded from Brazil [8–10], however, most of these records came from unpublished thesis and ecological studies [9], and several of these species were not found in recent taxonomic studies [11–14], rendering the real number of occurrences in the country difficult to access.

Brazilian specimens of *Branchiosyllis* cf. *exilis* (Gravier, 1900), *B*. *tamandarensis* **sp. n.,** *Haplosyllis lattigae* **sp. n.**, *H*. *loboi* Paola, San Martín & Martin, 2006, *Opisthosyllis brunnea* (Langerhans, 1879) and *O*. *viridis* Langerhans, 1879 are herein described, based on recently collected material from off the Brazilian coast. The cosmopolitan species *Trypanosyllis zebra* (Grube, 1860) is recorded for the first time for the states of Paraíba and Espírito Santo, whereas *Haplosyllis amphimedonicola* Paresque & Nogueira, 2014 and *H*. *rosenalessoae* Paresque & Nogueira, 2014 have their distribution expanded. Additionally, keys to the currently known species of *Branchiosyllis*, *Haplosyllis* and *Opisthosyllis* occurring along the Brazilian coast are provided.

## Material and Methods

### Field permit and deposit of specimens

Field Permit from Ministério do Meio Ambiente—MMA / Instituto Chico Mendes de Conservação da Biodiversidade—ICMBio Sistema de Autorização e Informação em Biodiversidade—SISBIO was gave to João Miguel de Matos Nogueira (number 1947272). Type material and voucher specimens are deposited at the Museu de Zoologia da Universidade de São Paulo, São Paulo, Brazil (MZUSP) (MZUSP 2858–2896) and Museo Nacional de Ciencias Naturales, Madrid, Spain (MNCN) (MNCN 1601/16877, 1601/16878, 1601/16879).

### Study area and sample processing

**Fig 1:**
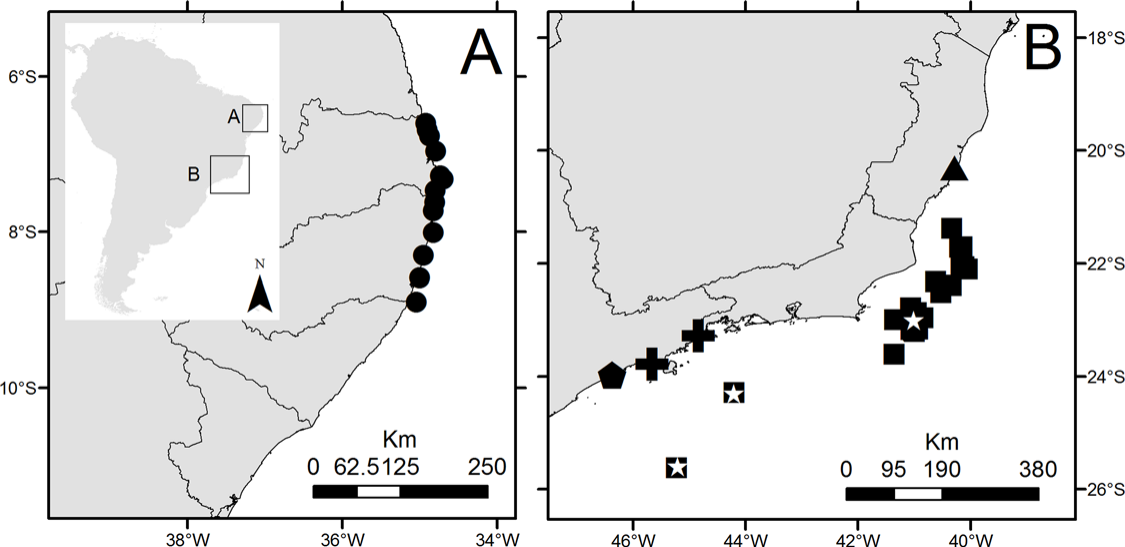
Map of distribution of the six different projects which collected the material used for the present paper: **A.** Northeastern Brazilian coast (● = collection sites from Project ‘Diversity of Polychaeta (Annelida) on sandstone reefs off northeastern Brazil, states of Paraíba and Pernambuco’); **B.** Southeastern Brazilian coast (▲ = ‘Influence of the fractal dimension of marine macroalgae on the associated community’; ■ = 'HABITATS Project–Campos Basin Environmental Heterogeneity by CENPES/PETROBRAS’; * = ‘REVIZEE/South Score/Benthos’; ➕ = ‘BIOTA/FAPESP/Benthic Marine Diversity in the State of São Paulo’; ⬟ = Diversity of Polychaeta (Annelida) on rocky shores off the State of São Paulo, southeastern Brazil).

The material examined for the present study came from six independent studies (Fig 1; Table 1). The first, project ‘Diversity of Polychaeta (Annelida) on sandstone reefs off northeastern Brazil, states of Paraíba and Pernambuco’ (‘*BioPol-NE*’*)*, was conducted by the Laboratório de Poliquetologia (LaPol), Instituto de Biociências, Universidade de São Paulo (IB/USP), with collections from the intertidal zone to ~1 m deep on sandstones, at neap tide, from reefs off the states of Paraíba and Pernambuco, northeastern Brazil. Algae, sponges, ascidians, mussel beds and similar substrates were scrapped from the rocks. In the laboratory, the material was examined alive under a stereomicroscope, polychaetes were sorted, relaxed in menthol solution, preserved in 4% formalin, and, a few weeks later, rinsed in fresh water and transferred to 70% ethanol.

**Table 1:** Sumary of the projects which provided material for the present study.

	1) BioPol-NE	2) Poly-Phytal	3) HABITATS	4) REVIZEE	5) BIOTA	6) BioPol-SP
Coordinates	6°36'S/8°54'S 35°8'W/34°48'W	20°18.466'S 40°16.728'W	21°23'S/25°37''S 40°10'W/45°14'W	23°10'S/25°37'S 40°56'W/45°14'W	23°23'S/23°58'S 44°50'W/46°11'W	23°59'S/24°1'S 46°19'W/46°22'W
Ecoregions from [75]	**75.** Northeastern Brazil	**76.** Eastern Brazil	**76.** Eastern Brazil and **180.** Southeastern Brazil	**76.** Eastern Brazil and **180.** Southeastern Brazil	**180.** Southeastern Brazil	**180.** Southeastern Brazil
Depth	intertidal to ~1 m	Intertidal	53−3301 m	153−800 m	Intertidal to ~80 m	Intertidal
Date	2010−2014	2005−2006	2009	1998	2001−2005	2002−2005
Sampling method	scrape	scrape	Van Veen grab	Van Veen grab	scrape	scrape
Biotope structures	Algae, sponges, ascidians, mussel beds and similar substrates	Algae	Sand	Sand	Sand and also algae, sponges, ascidians, mussel beds and similar substrates	Algae, sponges, ascidians, mussel beds and similar substrates

The project ‘Influence of the fractal dimension of marine macroalgae on the associated community’ (‘*Poly-Phytal*’), studied the fauna living on the algae *Arthrocardia gardneri* Manza, 1937, *Hypnea musciformis* (Wulfen) J. V. Lamouroux, 1813, *Sargassum* sp., *Centroceras clavulatum* (C. Agardh) Montagne, 1846, *Colpomenia sinuosa* (Mertens ex Roth) Derbès & Solier, 1851, and *Ulva fasciata* Delile, 1813. Algae were scraped from the rocks off Vitória, state of Espírito Santo, and preserved in 4% formalin. The solution was sieved through a 0.5-mm mesh; syllids were sorted and then rinsed in fresh water and transferred to 70% ethanol [15].

The project ‘HABITATS—Environmental Heterogeneity in the Campos Basin’ (‘*HABITATS*’) sampled in the Campos Basin, State of Rio de Janeiro, in a survey coordinated by the Brazilian energy company PETROBRAS. Collections were made from 12 to 3301 m deep in 2008 and 2009. Material was fixed in 4% formalin and later rinsed in fresh water and transferred to 70% ethanol.

The project ‘REVIZEE/Southern Score/Benthos’ (‘*REVIZEE*’) investigated the fauna occurring in the Brazilian Economic Exclusive Zone. Material was collected from 60 to 800 m deep, with dredges, box corers and Van Veen grabs (see [16] for collection details).

The project ‘BIOTA/FAPESP/Benthic Marine Biodiversity in the State of São Paulo’ (‘*BIOTA*’) studied the fauna occurring off the northern coast of São Paulo, with collections from the intertidal zone to ~45 m deep, on rocky shores, assemblages of algae and soft bottoms. For that study, material was preserved in 4% formalin immediately after collection, and specimens were posteriorly sorted out by family and transferred to 70% ethanol [17].

The sixth project, ‘Diversity of Polychaeta (Annelida) on rocky shores off the State of São Paulo, southeastern Brazil’ (‘*BioPol-SP*’*)* was also conducted by the LaPol, following the same methodology as project BioPol-NE, except for all collections having been made intertidally.

Further analyses under stereo- and light microscopes were made from specimens preserved in ethanol, some of which were permanently mounted on slides in glycerin jelly. For examination under scanning electron microscope (SEM), specimens were dehydrated in a series of increasingly stronger ethanol solutions, then critical point dried, covered with 25 nm of gold, and examined and photographed under the SEM at Laboratório de Microscopia Eletrônica, Instituto de Biociências, Universidade de São Paulo (IB/USP). Line drawings were made from slide-mounted specimens, with the aid of a drawing tube. Measurements were taken under compound and stereo microscopes. Length of the specimens was measured from the tip of palps to the tip of pygidium, excluding anal cirri; width was measured at proventricular level, excluding parapodia.

Comparative material was examined from specimens lodged at the MNCN, the Australian Museum, Sydney, Australia (AM), the Zoological Museum Hamburg, Germany (ZMH), United States National History Museum, Smithsonian Institution, Washington DC, USA (USNM) and National Museum of Nature and Science, Tsukuba, Japan (NSMT). The number of access of all material analysed in the present study can be found at sections "material examined" and additional material examined”along the text.

The nomenclature used for *Haplosyllis* chaetal morphology follows that suggested by [18] and [19]. The chaetal characters used here are:

- length of main fang: as long as, or longer than chaetal width;
- upper side of main fang: with or without denticles;
- mid-joining point between apical teeth and main fang: curved (usually short) or straight (at right angle with main fang, short or long);
- proximal and distal apical teeth: similar in size or one of them larger.

The nomenclature used for *Branchiosyllis* follows [20], in adopting 'ungula' (pl. 'ungulae') to name the modified, claw-shaped falcigers characteristic of this genus.

### Nomenclatural Acts

The electronic edition of this article conforms to the requirements of the amended International Code of Zoological Nomenclature, and hence the new names contained herein are available under that Code from the electronic edition of this article. This published work and the nomenclatural acts it contains have been registered in ZooBank, the online registration system for the ICZN. The ZooBank LSIDs (Life Science Identifiers) can be resolved and the associated information viewed through any standard web browser by appending the LSID to the prefix “http://zoobank.org/”. The LSID for this publication is: urn:lsid:zoobank.org:pub:6353431B-6724-42BC-9F54-D06CD5FA0F28. The electronic edition of this work was published in a journal with an ISSN, and has been archived and is available from the following digital repositories: PubMed Central, LOCKSS.

### Taxonomic Account

Family Syllidae Grube, 1850

Subfamily Syllinae Grube, 1850

Genus *Branchiosyllis* Ehlers, 1887

Type species: *Branchiosyllis oculata* Ehlers, 1887.

#### Diagnosis

Relatively medium-sized to large body, subcilindrical to dorso-ventrally or, more rarely, laterally flattened. Palps free or fused at bases. Prostomium with four eyes, occasionally also with two anterior eyespots, and three antennae. Peristomium with two pairs of peristomial cirri. Antennae, peristomial, dorsal and anal cirri articulated. Parapodial lobes sometimes with branchiae. Compound chaetae as falcigers with regular blades and with modified, claw-shaped blades, arranged in 90° with shaft ('ungulae'), at least on part of body. Simple chaetae not known, apparently altogether absent. Pharynx with single anterior tooth; proventricle usually of approximately same length as pharynx. Reproduction by acephalous stolon.

#### Remarks

The species of *Branchiosyllis* occurring along the American coasts were recently revised by [21], which reported *B*. *diazi* Rioja, 1958 and *B*. *exilis* (Gravier, 1900) for the Brazilian coast, *B*. *diazi* reported specifically from the State of Pernambuco, while *B*. *exilis* was considered as a circumtropical species [6]. In addition, [22] also recorded *B*. *diazi* from off the coast of Alagoas, and [12] registered the occurrence of *B*. *exilis* off the State of São Paulo. A third species, *B*. *oculata*, was recorded by [23] and [24] for Rio Grande do Norte, and by [22] and [25] for the State of Bahia.

### Identification key to the species of *Branchiosyllis* currently recorded in Brazilian waters

1a Branchiae absent… ***B*. *exilis***
1b Branchiae present… 2
2a.(1b) Bidentate and unidentate falcigers on anterior body, ungulae from midbody parapodia… ***B*. *diazi***
2b.(1b) Bidentate and unidentate falcigers absent, ungulae in all parapodia… **3**
3a.(2b) Proventricle through 3.5–5 chaetigers, with 25–30 muscle cells rows. Branchiae with up to 6 lobes… ***B*. *tamandarensis* sp. n.**
3b.(2b) Proventricle through 8 chaetigers, with 22 muscle cells rows. Branchiae dome shaped or slightly flattened… ***B*. *oculata***

*Branchiosyllis* cf. *exilis* (Gravier, 1900)

Figs 2–6

**Fig 2:**
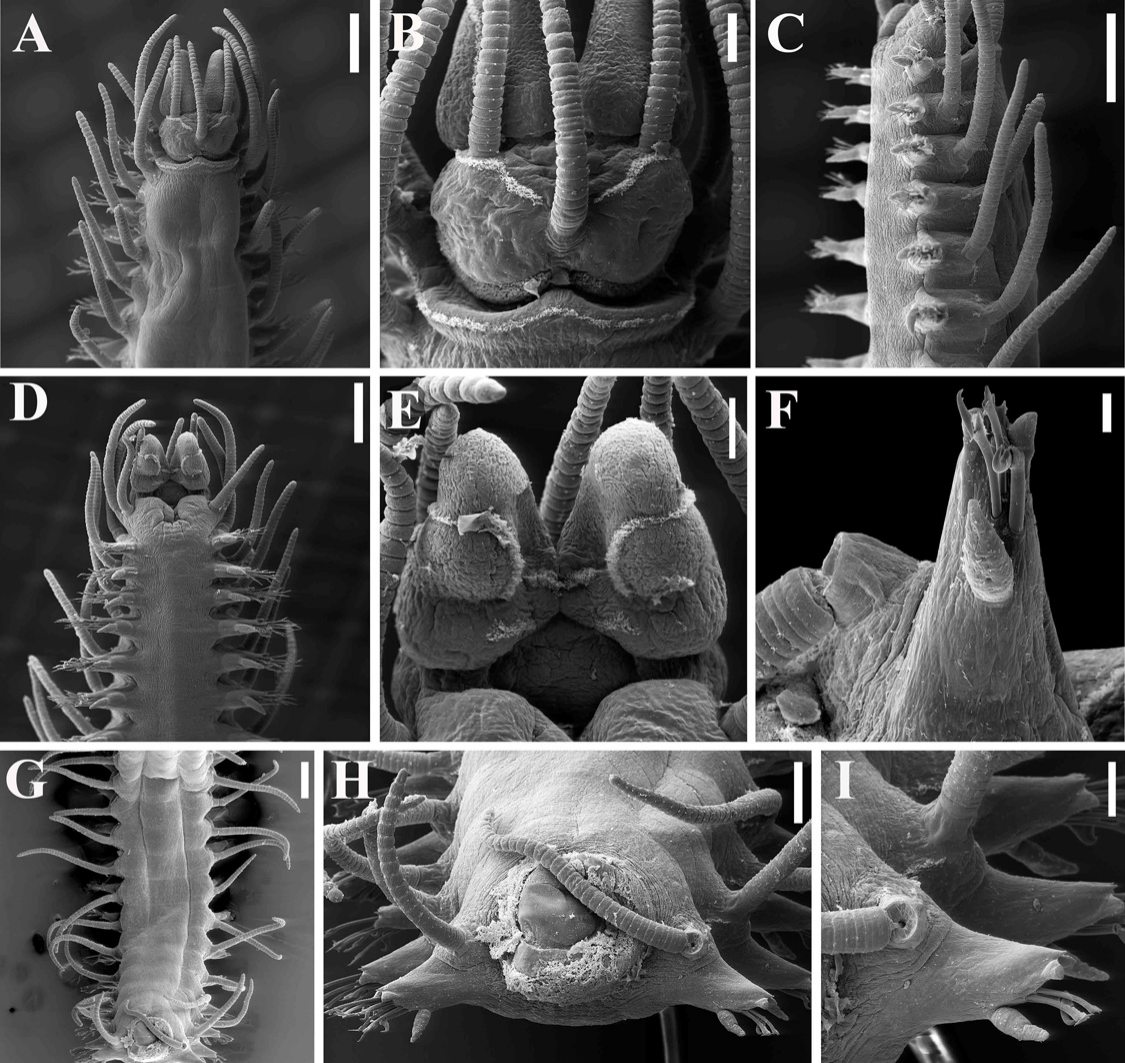
*Branchiosyllis* cf. *exilis*, SEM. (A) anterior body, dorsal view; (B) prostomium and peristomium, dorsal view; (C) anterior body, lateral view; (D) anterior body, ventral view; (E) detail of prostomium and peristomium, ventral view; (F) midbody parapodium, ventral view; (G) midbody segments, dorsal view; (H) midbody, rear view of end of fragment (incomplete specimen); (I) midbody parapodia. Scale bars: A, C–D, G, 200 μm; B, E, I, 50 μm; F, 20 μm; H, 100 μm.

**Fig 3:**
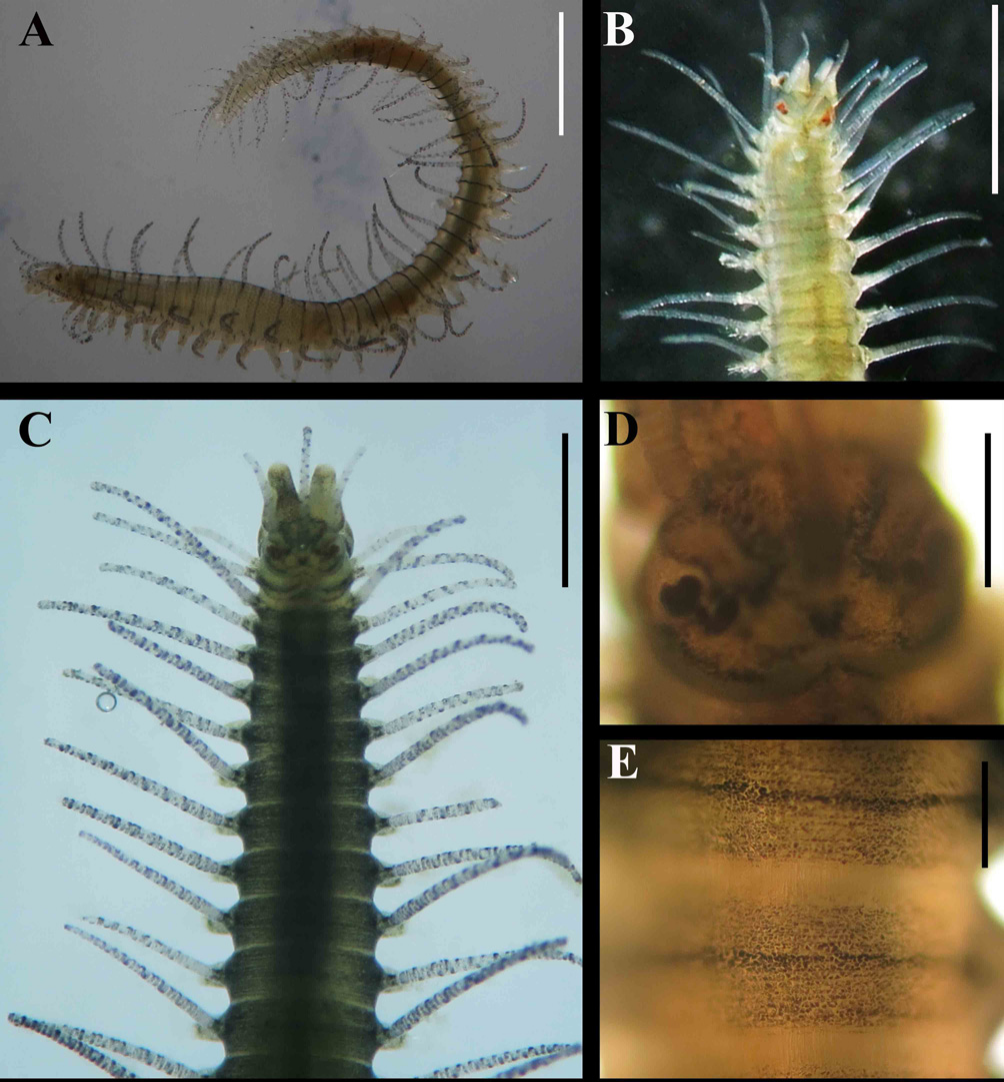
*Branchiosyllis* cf. *exilis*, live specimen. (A) complete specimen, lateral view; (B) (C) Anterior body, dorsal view; (D) prostomium, dorsal view; (E) midbody segments, dorsal view. Scale bars: A, B, 1 mm; C, 0.5 mm; D–E, 0.2 mm.

**Fig 4:**
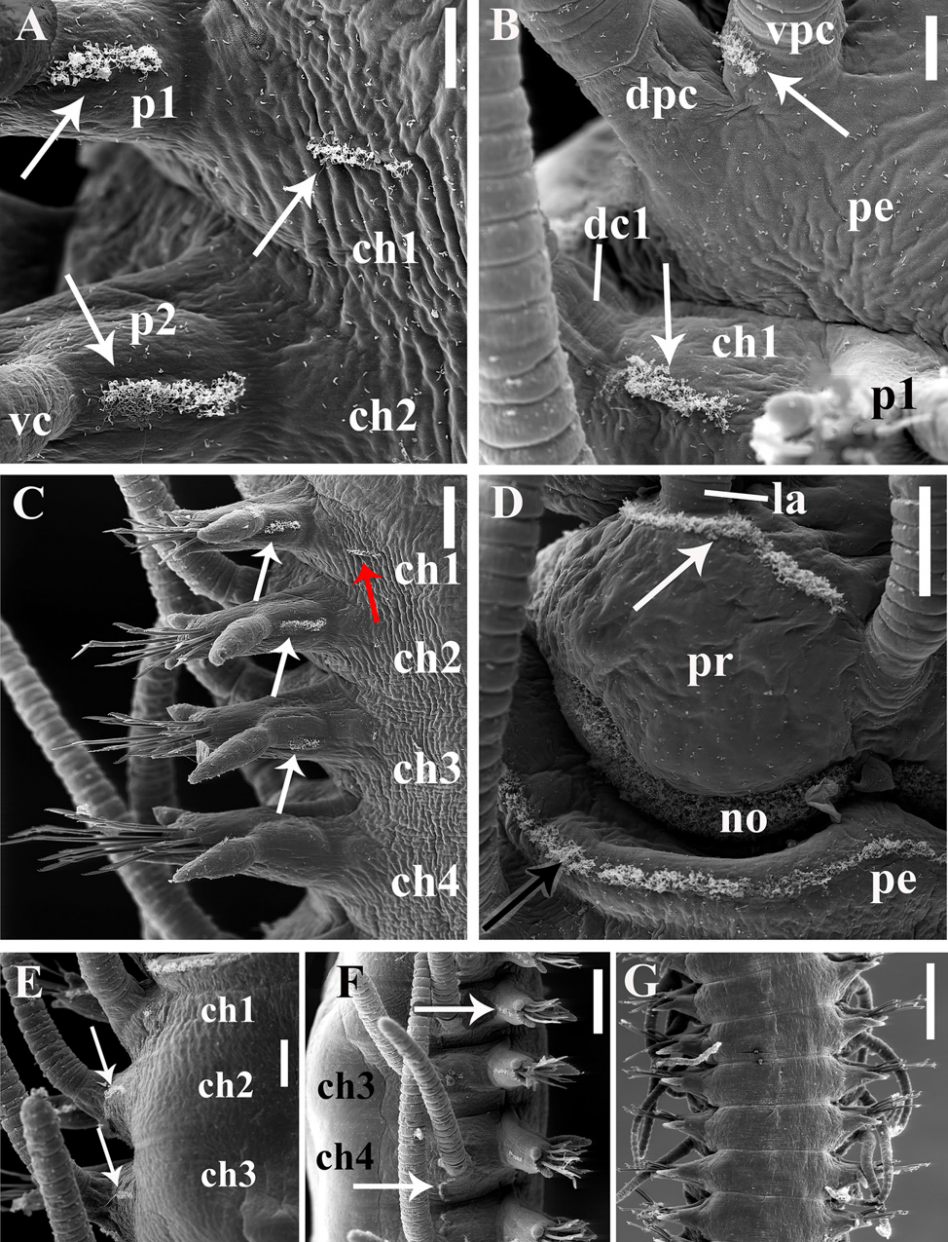
*Branchiosyllis* cf. *exilis*, SEM. (A) chaetigers 1–2, ventral view, arrows indicate tufts of cilia on chaetiger 1 and on parapodia of chaetigers 1 and 2; (B) detail of peristomium and chaetiger 1, ventral view, arrows indicate tufts of cilia on ventral peristomial cirrus and between parapodial lobe and dorsal cirrus of chaetiger 1; (C) chaetigers 1–4, ventral view, white arrows indicate tufts of cilia at bases of parapodia of chaetigers 1–3, red arrow indicate tuft of cilia on chaetiger 1; (D) detail of prostomium and peristomium, dorsal view, white arrow indicates row of cilia on prostomium, black arrow indicates row of cilia on peristomium; (E) chaetigers 1–3, dorsal view, arrows indicate tufts of cilia dorsally to bases of dorsal cirri; (F) anterior body, right lateral view, arrows indicate tufts of cilia dorsally to parapodium of chaetiger 2 and between dorsal cirri of chaetigers 4 and 5; (G) midbody segments, ventral view. **ch1–4**—chaetigers 1–4; **dc1—**dorsal cirrus, chaetiger 1; **dpc—**dorsal peristomial cirrus; **la—**lateral antenna; **no—**nuchal organ; **p1–2**—parapodia segments 1–2; **pe—**peristomium; **pr—**prostomium; **vc**—ventral cirrus; **vpc—**ventral peristomial cirrus. Scale bars: A, B, 20 μm; C–E, 50 μm; F, 100 μm; G, 200 μm.

**Fig 5:**
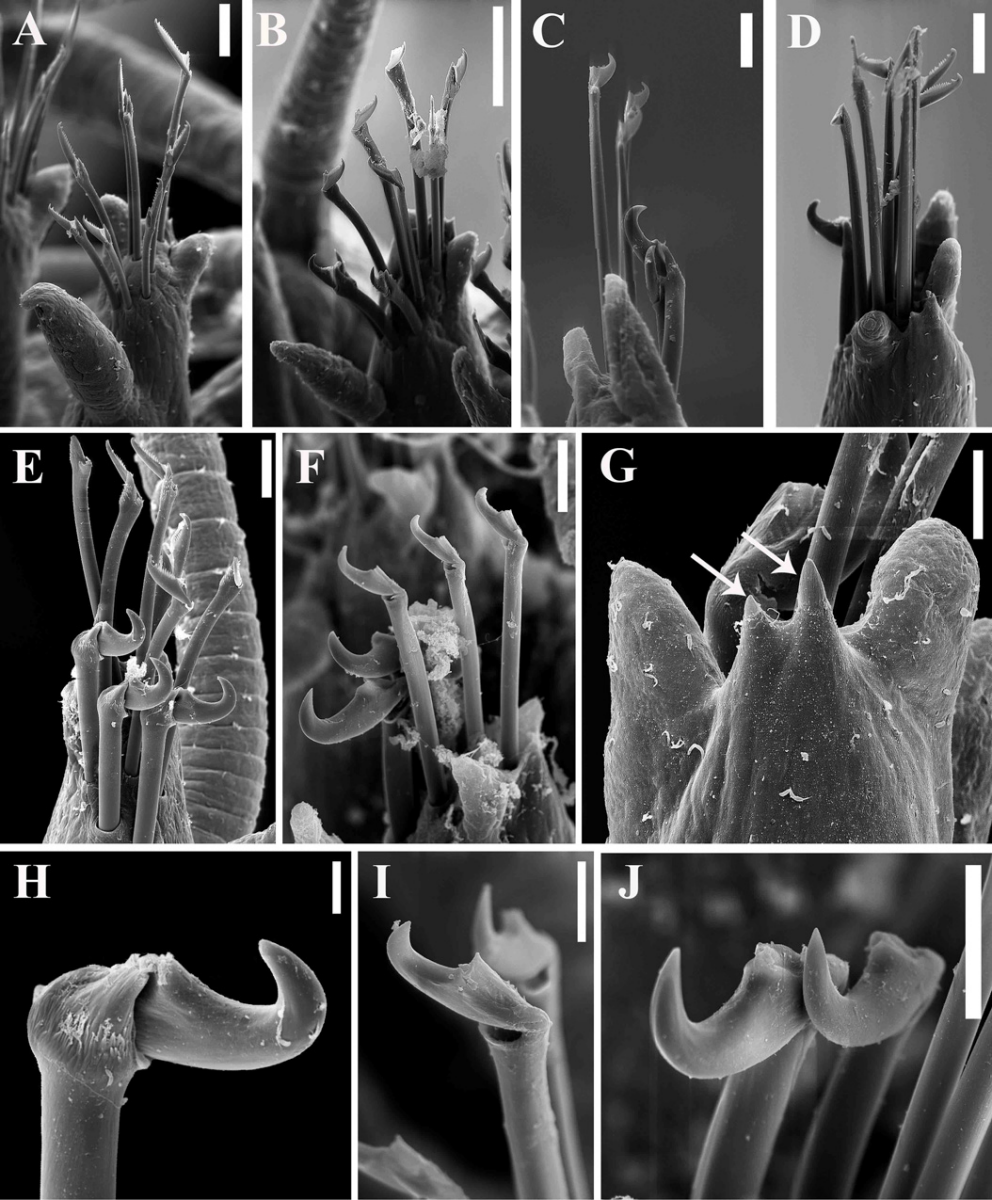
*Branchiosyllis* cf. *exilis*, SEM. (A) falcigers, parapodium 2, ventral view; (B) chaetae, parapodium 18; (C) chaetae, midbody parapodium; (D) chaetae, parapodium 28; (E) (F) chaetae, midbody parapodia; (G) detail of parapodium, dorsal view, arrows indicate protruding aciculae; (H) (I) (J) ungulae, mid- to posterior body chaetigers. Scale bars: A, 200 μm; B, 50 μm; C–F, J, 20 μm; G, I, 10 μm; H, 5 μm.

**Fig 6:**
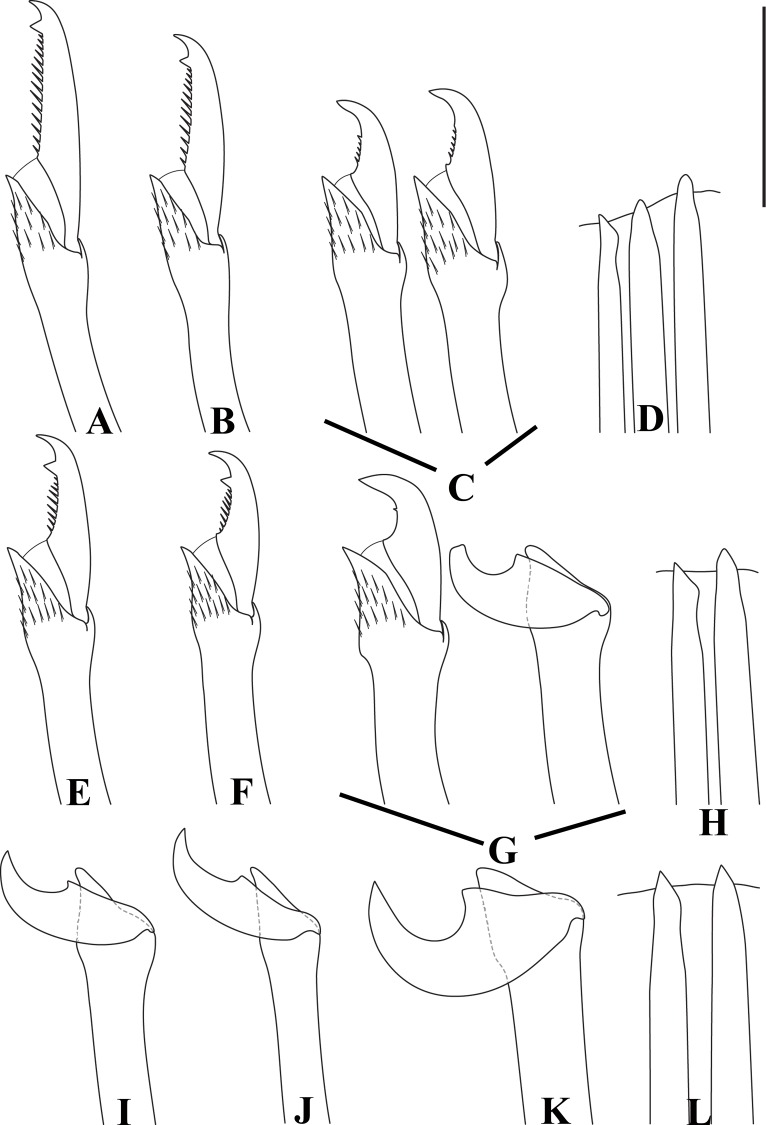
*Branchiosyllis* cf. *exilis*. (A) (B) (C) dorsal, intermediate and ventral falcigers, respectively, anterior body; (D) aciculae, anterior body; (E) (F) dorsal and intermediate falcigers, respectively, midbody; (G) ventralmost chaetae, midbody; (H) aciculae, midbody; (I) (J) (K) dorsal, intermediate and ventral ungulae, respectively, posterior body; (L) aciculae, posterior body. Scale bar: 30 μm.

*Branchiosyllis exilis*. San Martín, 1984: 294–303, lams. 69–72 [26]; 2003: 332–336, Figs 184–185 [2]; Nogueira, 2000: 98–99, Fig 22J–M [27]; 2006: 148 [12]; San Martín *et al*., 2008: 127–130, Figs 7, 8B–F [6].

#### Material examined

Project ‘*BioPol-NE*’. State of Paraíba: Mataraca, Barra de Camaratuba (06º36'S 34º57'W), intertidal: 2 specimens (MZUSP 2410), coll. 12 August 2010. Baía da Traição, Praia do Farol (06º41'S 34º55'W), intertidal: 1 specimen (MZUSP 2139), coll. 09 August 2010. Rio Tinto, Barra de Mamanguape (06º45'S 34º55'W), intertidal: 1 specimen (MZUSP 2141), coll. 11 August 2010. João Pessoa, Praia de Cabo Branco (07^o^08'S 34^o^47'W), intertidal, on sabellariid reefs: 1 specimen (MZUSP 2144), coll. 09 February 2009; 1 specimen (MZUSP 2143), coll. 02 February 2010. State of Pernambuco: Goiana, Pontas de Pedra (07°37'S 34°48'W), intertidal: 2 specimens (MZUSP 2142), coll. 13 December 2012. Sirinhaém, Barra do Sirinhaém (08°36.707'S 35°2.450'W), intertidal: 1 specimen (MZUSP 2208), coll. 23 July 2013.

#### Additional material examined

*Branchiosyllis lorenae* San Martín & Bone, 1999. Venezuela, Morrocoy Park, on *Thalassia testudinum* beds: holotype (MNCN 16.01/3704), coll. September 1993 and 3 paratypes (MNCN 16.01/3712), coll. December 1993, G. San Martín, det. G. San Martín & Bone.

*Branchiosyllis baringabooreen* San Martín, Hutchings & Aguado, 2008. Australia, South Australia, western side of Cassini Island (13°57'S 125°37'E), coralline algae and rubble: 1 paratype (AM W26511), coll. 18 July 1988, P.A. Hutchings, det. G. San Martín. Western Australia, Bernouli Island (15°00'S 124°47'E), sandy substrate with coral rubble, intertidal: holotype (AM W30088); 1 specimen (AM W30089), coll. 12 July 1988, P.A. Hutchings, det. G. San Martín. Western Australia, Lucas Island, Brunswick Bay (15°16'S 124°29'E), dead coral with *Sargassum* with heavy silt loading, 2 m: 2 specimens (AM W30090), coll. 24 July 1988, P.A. Hutchings, det. G. San Martín.

*Branchiosyllis carmenroldanae* San Martín, Hutchings & Aguado, 2008. Australia, Western Australia, Beacon Island, Goss Passage (28°25'30"S 113°47'E), on dead plates of *Acropora* sp., covered in coralline algae, 8 m: holotype (AM W30118), 3 paratypes (AM W30119), coll. 19 May 1994, P.A. Hutchings, det. G. San Martín.

*B*. *orbiniiformis* San Martín, Hutchings & Aguado, 2008. Australia, Western Australia, Lucas Island, Brunswick Bay (15°16'S 124°29'E), dead coral with *Sargassum* with heavy silt loading, 2 m: holotype (AM W30115), 2 paratypes (AM W30116), 1 specimen (AM W26512), coll. 24 July 1988, P.A. Hutchings, det. G. San Martín.

*Branchiosyllis thylacine* San Martín, Hutchings & Aguado, 2008. Australia, New South Wales, 50 m west of Split Solitary Island (30°14'S 153°10'48"E), on *Herdmania momus*, rocks, sponges and ascidians, 16 m: holotype (AM W30120), 2 paratypes (AM W30121), coll. 7 Mar. 1992, P.A. Hutchings & C.L. Rose, det. G. San Martín.

#### Description

Mid-sized body, longest specimen analysed ca. 15.5 mm long, 0.7 mm wide, with 66 chaetigers; body subcylindrical (Figs 2C; 3A; 4F). Live specimens with one dark, transverse stripe per chaetiger along body, at midlength of a broader difuse dark band, and also a prostomial mask and dark spots on antennae and cirri throughout (Fig 3A–3E). Palps triangular, distally rounded, fused at bases (Figs 2A, 2D and 2E; 3B and 3C), each palp with three rows of cilia: longer row beginning on prostomium, anteriorly to base of median antenna, running towards bases of lateral antennae and then into palps, C-shaped ventrally, on middle third of palps (Figs 2A and 2B, 2D and 2E; 4D); remaining rows transverse, ventral and distinctly shorter, one near posterior margin of palps, laterally to mouth, another at point of fusion between palps (Fig 2E). Prostomium subpentagonal, shorter than palps, with two pairs of red eyes in open trapezoidal arrangement, anterior eyespots absent (Fig 3B–3D); median antenna inserted between posterior eyes, elongated, reaching beyond tip of palps, with ca. 17–28 articles (Figs 2A and 2B; 3B and 3D; 4D); lateral antennae slightly shorter than median antenna, inserted at anterior margin of prostomium, with 12–23 articles each (Figs 2A; 3B–3D; 4D). Ciliated nuchal organs between prostomium and peristomium, only visible under SEM (Fig 4D). Peristomium slightly shorter than subsequent segments, with small anterior lobe and a row of cilia on anterior margin (Figs 2A and 2B; 4D); dorsal peristomial cirri longer than antennae, with 28–34 articles each; ventral peristomial cirri shorter, about as long as lateral antennae, with 14–23 articles each, with tuft of cilia on posterior side of cirrophore, between peristomial cirri (Fig 4B). Dorsal cirri of chaetiger 1 as long as dorsal peristomial cirri, with 33–47 articles each; dorsal cirri of chaetigers 2 and 5 about same size, with 21–31 and 22–35 articles each, respectively; dorsal cirri of chaetiger 3 longer, with 28–41 articles each; dorsal cirri of chaetiger 4 shorter, with 29–45 articles each; following dorsal cirri alternating long and short (Fig 2G and 2H); two rows of cilia at bases of dorsal cirri, one dorsally and another posteriorly (Fig 4E and 4F). Chaetiger 1 with additional pair of tufts of cilia ventrally (Fig 4A). Antennae, peristomial and dorsal cirri throughout with cirrophores (Fig 2F and 2I). Ventral cirri ovate to pyriform, inserted at midlength of parapodial lobes, reaching their tips (Figs 2F, 2H and 2I; 4C and 4G); row of cilia at base of ventral cirri (Fig 4C). Parapodial lobes elongate, distally bilobed (Figs 2F, 2H and 2I; 4C, 4F and 4G; 5G) with digitiform, similar in length prechaetal and postchaetal lobes (Fig 5G); at least three anteriormost parapodia with short, transverse row of cilia dorsally (Fig 4A and 4F), and another ventrally to bases of ventral cirri (Fig 4C), absent from chaetiger 4–5 (Fig 4C). Branchiae absent. Anteriormost parapodia with 5–8 falcigers each (Fig 5A); parapodia from proventricle level with up to 4 uni- to subbidentate and 4–12 bidentate falcigers each, ungulae absent (Figs 5B; 6A–6C); each midbody parapodium with three types of chaetae, 1–6 bidentate falcigers, 2–6 uni- to subbidentate falcigers and 1–5 ungulae (Figs 5C–5F; 6E–6G); number of bidentate falcigers per parapodium decreasing and number of uni- to subbidentate falcigers and ungulae per parapodium increasing towards posterior body; posterior parapodia with 2–6 ungulae each (Figs 5H–5J; 6I–6K), falcigers absent. Shafts of falcigers subdistally spinulated (Fig 6A–6C, 6E–6G); blades of uni- and subbidentate chaetae smooth to slightly spinulated (Fig 6C and 6G); blades of bidentate falcigers spinulated, teeth similar in length or subdistal tooth slightly shorter (Fig 6A, 6B and 6E); blades of anterior and midbody falcigers with dorso-ventral gradation in length, 42–25μm long and 28–20μm long on anterior and midbody parapodia, respectively; ventralmost ungulae on midbody parapodia 20–23 μm long; blades of ungulae smooth (Figs 5H–5J; 6G, 6I–6K), ventralmost 1–2 ungulae larger than remaining in each parapodium posteriorly (Fig 5E and 5F), 25–30 μm long. Anterior parapodia with up to 3 aciculae each, one of which irregularly inflated subdistally, remaining aciculae straight, with acute tip (Fig 6D); mid- and posterior body parapodia with up to 2 aciculae each, subdistally inflated, distally pointed (Fig 6H and 6L), tips of aciculae frequently protruding from parapodial lobes throughout (Fig 5G). Pygidium semicircular, with pair of elongated anal cirri, about same length as posterior dorsal cirri. Pharynx through 4–8 segments; tooth slightly away from anterior border; proventricle extending for 6–8 chaetigers, with ca. 42 rows of muscle cells.

#### Remarks

*Branchiosyllis exilis* is a cosmopolitan species, which was reviewed recently by [28], who analysed morphologically a great number of specimens from several localities around the world and divided them in two morphological groups. The authors concluded that a molecular approach is necessary to determine if there is a single species, with slight differences among populations, or if there are several sibling species grouped together under the name *Branchiosyllis exilis*. Considering this, Brazilian specimens of *Branchiosyllis* cf. *exilis* fit the group of the holotype (and material from Australia and Cuba), with slender and elongate cirri (longer than body width, 30–40 articles), blades of falcigers with dorso-ventral gradation in length and with both teeth similar in size. About the colour pattern, they noticed this character to be highly variable and thus not useful for identification, at least in preserved specimens. The Brazilian material matches the description provided by [28], although with slight differences on dorsal pigmentation. Considering this variation, we treated the Brazilian specimens as *Branchiosyllis* cf. *exilis*.

*Branchiosyllis lorenae*, *B*. *salazari*, *B*. *maculata* (Imajima, 1966) and *B*. *thylacine* San Martín, Hutchings & Aguado, 2008 also lack branchiae, have ungulae starting from midbody chaetigers and dark spots on cirri, similar to the pattern found in *B*. cf. *exilis*. The first two species are known from the Caribbean, while the remaining species are both from the Pacific [28]. *Branchiosyllis lorenae* differs from *Branchiosyllis* cf. *exilis* by having three ovate spots dorsally on each segment, except for the anteriormost chaetigers; peristomium dorsally reduced; falcigers with subdistal tooth shorter than distal tooth; and ungulae present only on posterior parapodia. Brazilian specimens of *Branchiosyllis* cf. *exilis* have a different colour patern, as a transverse dorsal stripe per chaetiger, sometimes with additionally diffused dark band dorsal and ventrally; peristomium dorsally conspicuous, although shorter than following chaetigers; and ungulae starting from midbody.

*Branchiosyllis salazari* was described by [29] in a paper which also analysed the type material of *B*. *exilis*. The authors noticed that the holotype of *B*. *exilis* does not have dorsal pigmentation, however, as already mentioned, this character is extremely variable, especially considering preserved material [28]. *Branchiosyllis salazari* and *B*. *exilis* are distinguished from each other mostly based on the morphology of anterior and midbody aciculae, and also on the size of the teeth of midbody falciger blades. According to description provided by [29], *B*. *salazari* has three aciculae per parapodium anteriorly, two of which straight, with acute tips, the remaining subdistally oblique, with acute tip; from midbody, each parapodium has two aciculae, one of which straight, with acute tip, the other subdistally oblique, with tip directed upwards (Fig 3B, D in [29]); on the other hand, according to [29], *B*. *exilis* has two aciculae per parapodium anteriorly, one L-shaped and another subdistally oblique, and posterior parapodia with single acicula each, distally tapering and curved. Furthermore, the blades of falcigers on midbody parapodia are bidentate in *B*. *exilis*, with distal tooth ca. three times larger than the subdistal one (at least in ventralmost chaetae), while midbody falciger blades of *B*. *salazari* are unidentade [29]. Also, live specimens of *B*. *salazari* have different pattern of dorsal pigmentation when compared with live material of Brazilian *Branchiosyllis* cf. *exilis* (Leslie Harris, personal communication). According to an unpublished photo by Leslie Harris, *B*. *salazari* has anterior chaetigers with two brownish, dimmed, transverse bands per chaetiger, one discontinuous, mid-dorsal with lateral gaps, and another near posterior border of each chaetiger.

Brazilian specimens of *Branchiosyllis* cf. *exilis* differ from the description of the holotype provided [29] by having ventralmost falcigers subbidentate to unidentate and lacking L-shaped aciculae.

*Branchiosyllis maculata* differs from *Branchiosyllis* cf. *exilis* by having some completely black articles on dorsal cirri throughout, dorsum lacking pigmentation patterns, longer dorsal cirri throughout, and ungulae only present on posteriormost parapodia. On the other hand, *Branchiosyllis* cf. *exilis* does not have black articles on dorsal cirri, although some scattered black spots are common. Finally, *B*. *thylacine* can be differentiated from Brazilian specimens of *Branchiosyllis* cf. *exilis* by only having ungulae on posterior parapodia, all falcigers with unidentate blades and different aciculae, straight and with rounded tips.

#### Type locality

Red Sea (Indian Ocean).

#### Distribution

Apparently circumtropical, also present in the warmest regions of the Mediterranean sea [28]. First record from off the northeastern Brazilian coast.

*Branchiosyllis tamandarensis* sp. n.

**Fig 7:**
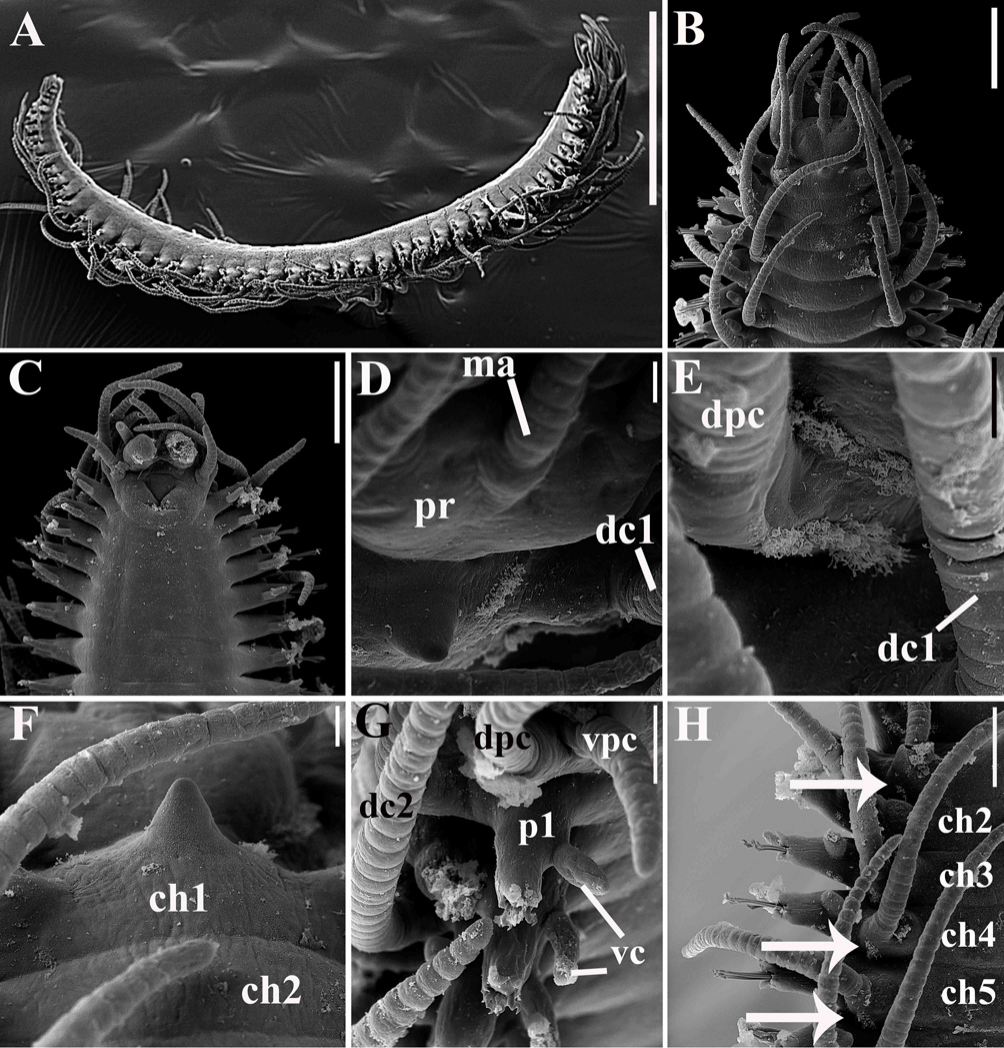
*Branchiosyllis tamandarensis* sp. n., SEM. (A) complete specimen, ventro-lateral view; (B) (C) anterior body, dorsal and ventral views, respectively; (D) detail of anterior body, fronto-dorsal view; (E) detail of peristomial ciliation, dorsal view; (F) detail of chaetiger 1, dorsal view; (G) anterior parapodia, lateral view; (H) anterior parapodia, dorsal view, arrows indicate tufts of cilia on posterior side of base of dorsal cirri. **ch1–5**—chaetigers 1–5; **dc1–2—**dorsal cirri, chaetigers 1–2; **dpc—**dorsal peristomial cirrus; **ma—**median antenna; **pr—**prostomium; **vc**—ventral cirri; **vpc—**ventral peristomial cirrus. Scale bars: A, 1 mm; B–C, 200 μm; D–F, 20 μm; G, 50 μm; H, 100 μm.

**Fig 8:**
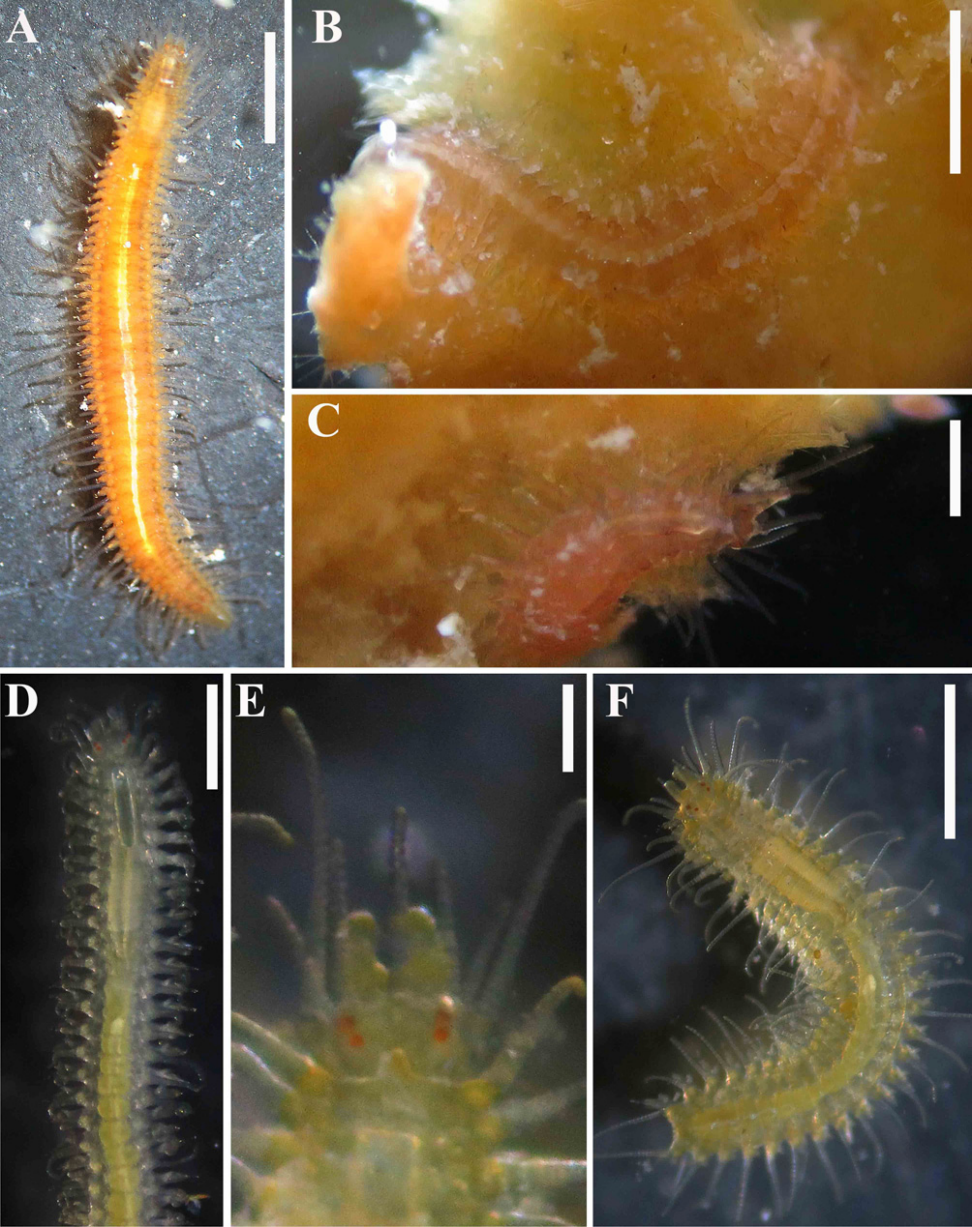
*Branchiosyllis tamandarensis* sp. n., live specimen. (A) complete specimen, dorsal view; (B) (C) specimens in sponge *Tedania ignis*; (D) (E) (F) anterior body, dorsal views. Scale bars: A–B, D, F, 1 mm; C, 0.5 mm; E, 200 μm.

**Fig 9:**
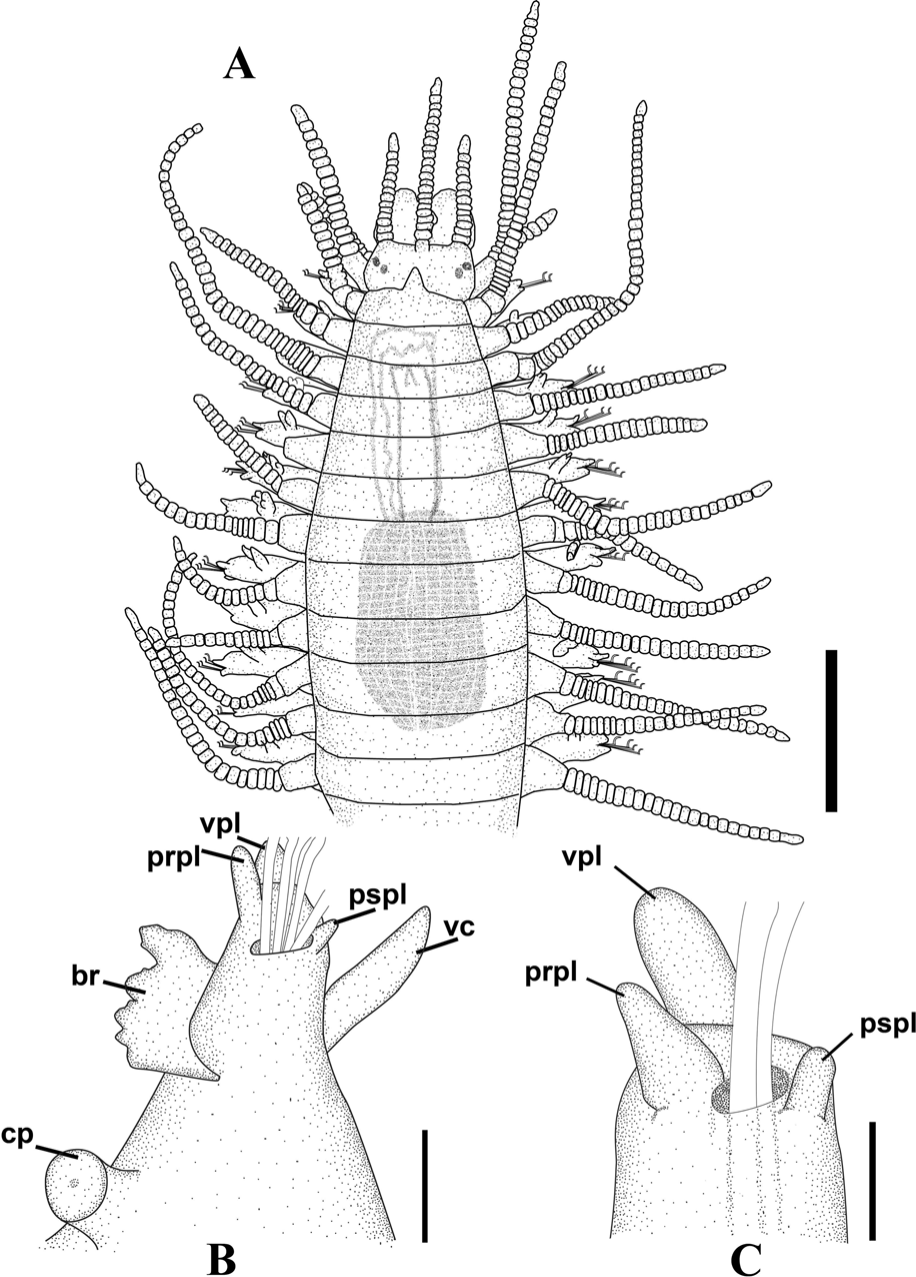
*Branchiosyllis tamandarensis* sp. n. (A) anterior body, dorsal view; (B) midbody parapodium, lateral view; (C) detail of midbody parapodial lobe, dorsal view. **br**—branchia; **cp**—cirrophore; **prpl**—prechaetal parapodial lobe; **pspl**—poschaetal parapodial lobe; **vc**—ventral cirrus; **vpl**—ventral parapodial lobe. Scale bars: A, 500 μm; B, 40 μm; C, 20 μm.

**Fig 10:**
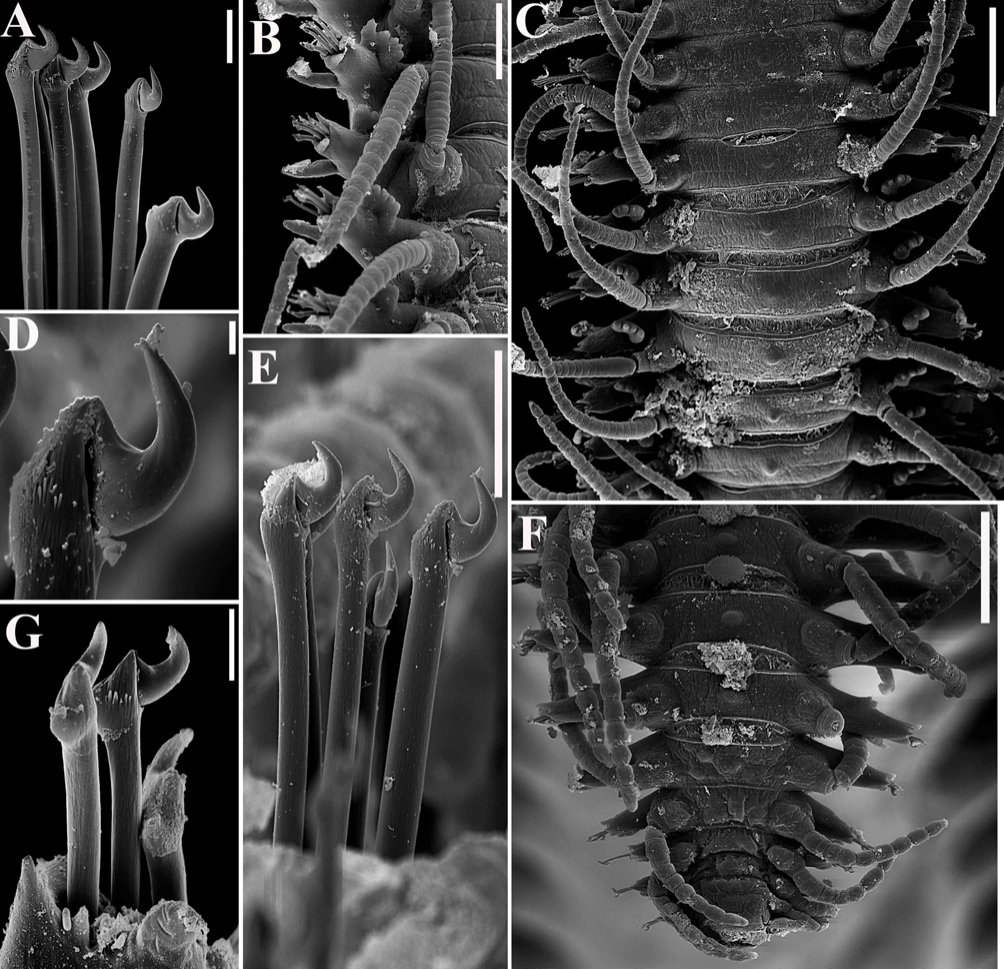
*Branchiosyllis tamandarensis* sp. n., SEM. (A) ungulae, parapodium 5; (B) midbody parapodia, dorsal view; (C) midbody segments, dorsal view; (D) (E) ungulae, midbody; (F) posterior segments, dorsal view; (G) ungulae, posterior body. Scale bars: A, 10 μm; B, 100 μm; C, 200 μm; D, 2 μm; E, 20 μm; F, 5 μm; G, 100 μm.

**Fig 11:**
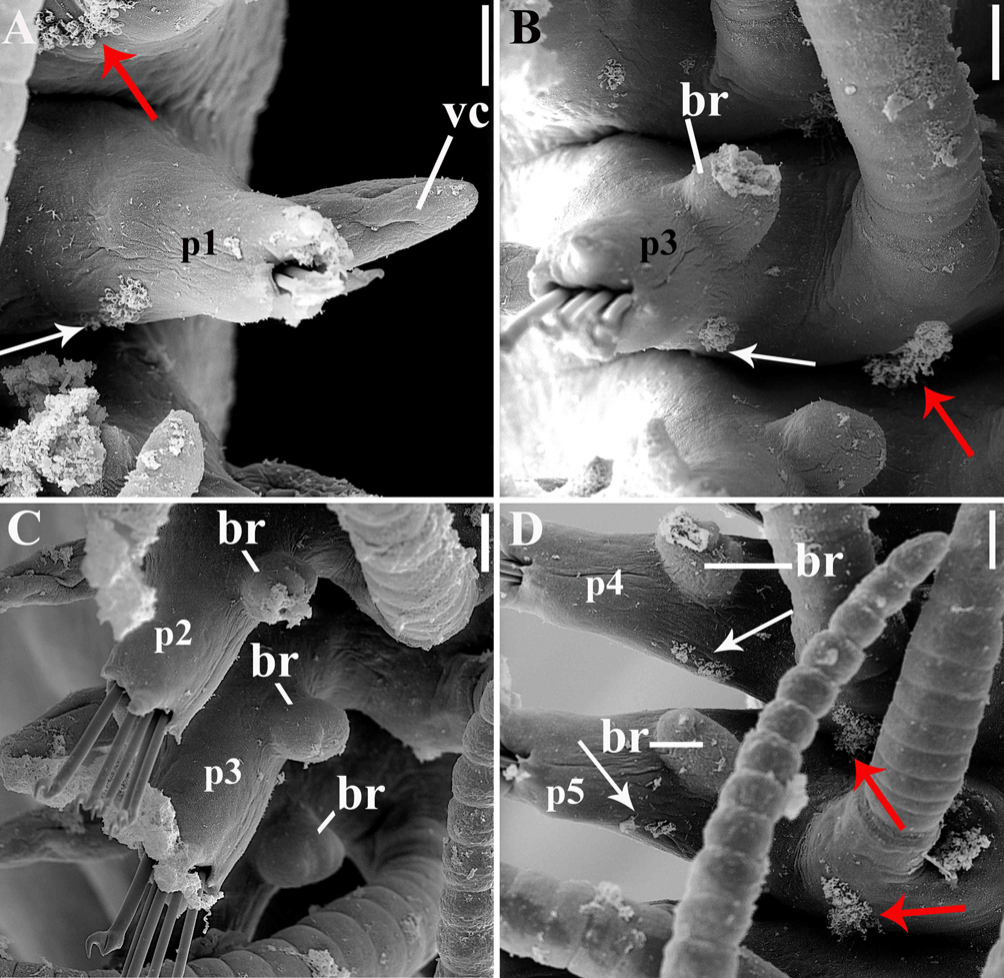
*Branchiosyllis tamandarensis* sp. n., SEM. (A) parapodium 1, dorso-lateral view; (B) parapodium 3, dorso-lateral view; (C) anterior parapodia, dorso-lateral view; (D) anterior parapodia, dorsal view. White arrows indicate tufts of cilia on posterior side of parapodia, red arrows indicate tufts of cilia on posterior side of bases of dorsal cirri. **br—**branchia; **p1–p5**—parapodium 1–5; **vc—**ventral cirrus. Scale bars: A–D, 20 μm.

**Fig 12:**
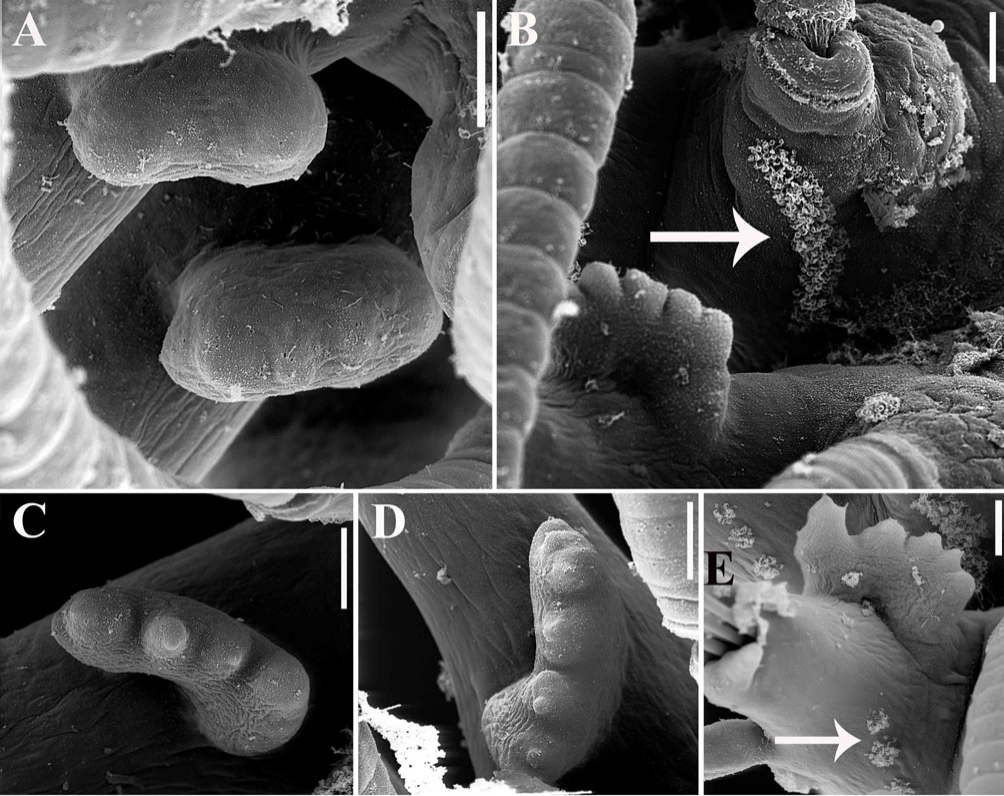
*Branchiosyllis tamandarensis* sp. n., SEM. (A) branchiae, parapodia 4–5; (B) branchia, midbody parapodium, arrows indicate tufts of cilia on posterior side of base of dorsal cirrus; (C) (D) (E) branchiae, midbody parapodia, arrow in E indicates tufts of cilia on posterior side of parapodia. Scale bars: A–E, 20 μm.

**Fig 13:**
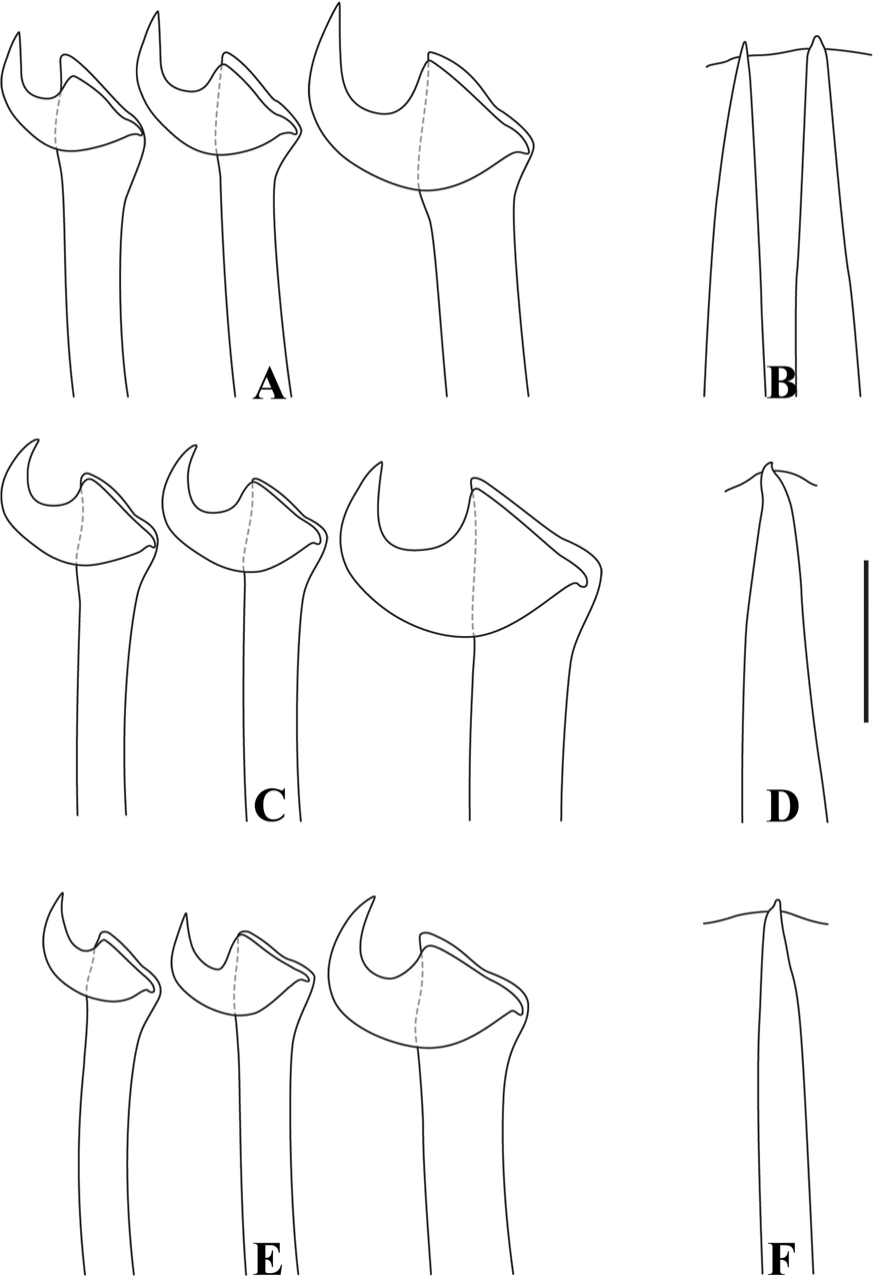
*Branchiosyllis tamandarensis* **sp. n.** (A) (C) (E) ungulae, anterior, mid- and posterior body, respectively; (B) (D) (F) aciculae anterior, mid- and posterior body, respectively. Scale bar: 15 μm.

Figs 7–13; Tables 2 and 3

urn:lsid:zoobank.org:act:71489F78-89CF-4640-99C9-BA9BFC84B111

#### Material examined

Project ‘*BioPol-NE*’. State of Paraíba: Baía da Traição, Praia do Farol (06º41'S 34º55'W), intertidal: 1 specimen (MZUSP 2134), coll. 09 August 2010. Rio Tinto, Barra de Mamanguape (06º45'S 34º55'W), intertidal: 26 specimens, coll. 11 August 2010. João Pessoa, Praia de Cabo Branco (07º 08'S 34º 47'W), intertidal: 2 specimens (MZUSP 2136), coll. 02 February 2010; on rhodolith: 1 specimen (MZUSP 2137), coll. 09 February 2009. State of Pernambuco: Goiana, Pontas de Pedra (07°37'S 34°48'W), intertidal: 1 specimen (MZUSP 2138), coll. 13 December 2012. Sirinhaém, Barra do Sirinhaém (08°36.707'S 35°2.450'W), intertidal: 2 specimens (MZUSP 2133), coll. 23 July 2013. Tamandaré, Praia dos Carneiros (08°42.849'S 35°4.999'W), intertidal, in *Tedania ignis* (Duchassaing & Michelotti, 1864): 1 specimen (MZUSP 2132), coll. 22 July 2013.

#### Type series

All specimens collected by the Project '*BioPol-NE*'. State of Pernambuco, Tamandaré, Praia dos Carneiros (08°42.849'S 35°4.999'W), intertidal: Holotype (MZUSP 2858), Paratypes 1 and 2 (MZUSP 2859–2860) coll. 22 June 2013; Sirinhaém, Barra do Sirinhaém (08°36.707'S 35°2.450'W), intertidal: Paratype 3 (MNCN 16.01/16877), coll. 23 June 2013. State of Paraíba: Rio Tinto, Barra de Mamanguape (06º45'S 34º55'W), intertidal: Paratype 4 (MNCN 16.01/16878), coll. 11 August 2010. Morphological data of specimens of the type series is provided in Table 2.

**Table 2:** Morphological variation among selected specimens of the type series of *Branchiosyllis tamandarensis* sp. n.

	MZUSP 2858	MZUSP 2859	MZUSP 2860	MNCN 16.01/16877	MNCN 16.01/16878
	Holotype	Paratype 1	Paratype 2	Paratype 3	Paratype 4
Number of chaetigers / Total length x width at proventricle (mm)	53 / 6 x 0.8	52 / 5.3 x 0.6	52 / 5.7 x 0.7	46 / 4.1 x 0.6	55 / 6 x 0.7
Length of pharynx (chaetigers)	4	4	5	5	5
Length of proventricle (chaetigers) / number of muscle cell rows	3.5 / 27	6 / 30	4.5 / 26	5 / 28	5 / 27
**Number of articles**					
Median antennae	18	19	17	18	17
Lateral antennae	17, 19	17,16	16,?	17, 17	16,?
Dorsal peristomial cirri	30, 19 (inc.)	?	27, 26	29, 27	28, 27
Ventral peristomial cirri	14, 15	17,	12,?	12,?	12–13
Dorsal cirri 1	22, 18 (inc.)	?	27, 21 (inc.)	27, 25	30, 29
Dorsal cirri 2	24, 26	?	21,?	25, 24	24,25
Dorsal cirri 3	27, 24	38, 39	31,?	26, 27	36,?
Dorsal cirri 4	21, 17	29,?	22,?	20,?	27,?
Dorsal cirri 5	22, 24	30,?	23,?	22, 23	23,?
Long cirri, midbody	25–27	23–30	20–27	22–24	27–29
Short cirri, midbody	12–17	18–21	15–17	13–18	18–21
Anal cirri	?	?	?	?	
**Number of ungulae per parapodium**					
Anterior body	2–6	2–6	2–6	2–6	2–5
Midbody	2–4	4–5	3–5	3–4	2–5
Posterior body	2–4	2–4	2–4	2–4	2–4
**Number of aciculae per parapodium**					
Anterior body	2	2	2	2	2
Midbody	2	1	1	1	1–2
Posterior body	1	1	1	1	1
Remarks					Gametes from chaetiger 13

#### Additional material examined

*Branchiosyllis oculata* Ehlers, 1887. Cuba, Archipiélago de los Canarreos, Cayos Bocas de Alonso, Canal de los Vapores: 9 specimens (MNCNM 16.01/752) coll. March 1990, coll. & det. G. San Martín.

*Branchiosyllis pacifica* Rioja, 1941. Panamá, Isla del Canal de Afuera (07º41'50''N 81º38'25W), 6 m: 10 specimens (MNCN 16.01/11586), coll. 12 February 1997, coll. & det. M. Capa; Isla Jicarita (07º37'50''N 81º44'30''W), 12 m: 1 specimen (MNCN 16.01/11725), coll. 9 February 1997, coll. & det. M. Capa; Coiba National Park: Isla de Uvas (07º49'00''N 81º46'00'W), coral rubble, 3 m: 18 specimens (MNCNM 16.01/11585), 11 specimens (MNCN 16.01/11588), coll. 6 February 1997, coll. & det. M. Capa.

#### Description

Relatively small-sized body, longest specimen analysed ca. 6 mm long, 0.5 mm wide, with 55 chaetigers (Table 2); body dorsoventrally flattened, ribbon-like (Fig 7A);. Live specimens yellow to orange (Fig 8A–8F). Reniform, distally rounded palps, fused at bases (Figs 7B and 7C; 8E; 9A). Prostomium pentagonal to ovate, about as long as palps, with two pairs of eyes in trapezoidal arrangement, anterior eyespots absent (Figs 8D–8F; 9A); median antenna inserted between or slightly anteriorly to anterior pair of eyes, elongated, reaching beyond tip of palps, with 17–19 articles (Figs 7B; 8E; 9A); lateral antennae inserted on anterior border, slightly shorter than median antenna, with 16–19 articles each (Figs 7B; 8E; 9A; Table 2). Peristomium dorsally inconspicuous, covered by a fold of chaetiger 1 (Figs 7D, 7F; 8E; 9A); dorsal peristomial cirri longer than antennae, with 19–30 articles each; ventral peristomial cirri shorter, about as long as lateral antennae, with 12–17 articles each (Table 2); bases of dorsal peristomial cirri with row of cilia (Fig 7E). Chaetiger 1 with projection over prostomium at dorsal midline, almost reaching level of posterior pair of eyes (Figs 7D, 7F; 8E; 9A) with paired tufts of cilia laterally (Fig 7D and 7F). Dorsal cirri of chaetiger 1 shorter than dorsal peristomial cirri, with 22–30 articles each; dorsal cirri of chaetiger 2 short, with 22–26 articles each; dorsal cirri of chaetiger 3 longest, with 24–39 articles each; dorsal cirri of chaetiger 4 shorter, with 17–29 articles each; following dorsal cirri alternating long, with 20–30 articles each, longer than body width, and short, with 12–21 articles each, shorter than body width at corresponding segment; bases of dorsal cirri with longitudinal row of cilia posteriorly (Figs 7H; 11B and 11D; Table 2). Antennae, peristomial and dorsal cirri throughout with cirrophores (Figs 9B, 10B and 10C). Digitiform ventral cirri, inserted at midlength of parapodial lobes or slightly closer to base, reaching tips of lobes (Figs 7C, 7G; 9B). Parapodial lobes elongate, distally trilobed (Figs 7G and 7H; 9B and 9C; 11A–11C), with digitiform to piriform prechaetal lobe, distinctly longer than postchaetal and similar in length or slightly shorter than ventral lobe (Fig 9B and 9C); single branchia on each parapodium, located dorsally, starting from chaetiger 2; branchiae bi- or trilobed on anterior and posterior parapodia, with up to six lobes from midbody parapodia (Figs 9B; 10B; 11B–11D; 12A–12E); parapodial lobes with posterior tuft of cilia (Fig 11A and 11B and 11D). Parapodium 1 with two ungulae each, from parapodium 2, anterior parapodia with 4–6 ungulae each; midbody parapodia with 2–5, posterior parapodia with 2–4 ungulae each (Table 2); falcigers absent throughout. Shafts of ungulae triangular, slightly spinulated subdistally, spinulation only visible under SEM (Fig 10A, 10D and 10E and 10G); ungulae with smooth, strongly curved, unidentate blades (Figs 10A, 10D and 10E, 10G; 13A, 13C and 13E); each parapodium with 1–2 ventralmost chaetae larger than remaining (Fig 13A, 13C and 13E); blades 13–25 μm long on anterior parapodia, 15–30 μm and 12–22 μm long on mid- and posterior body parapodia, respectively. Anterior parapodia with two aciculae each, straight, with acute tip, tips protruding from parapodial lobe (Fig 13B); mid- and posterior body parapodia with single acicula each, distally slightly sinuous, with acute tip (Fig 13D and 13F). Pygidium semicircular, with two elongated anal cirri, as long as posterior dorsal cirri. Pharynx through 4–5 segments, tooth at anterior border (Fig 9A); proventricle extending for 3.5–6 chaetigers, with 26–30 rows of muscle cells.

#### Remarks

*Branchiosyllis tamandarensis* **sp. n.** resembles *B*. *oculata*, *B*. *pacifica* and *B*. *lamellifera* by having flattened, ribbon-like body, branchiae present, ungulae on all parapodia, and falcigers absent (Table 3). *Branchiosyllis oculata* and *B*. *lamellifera* were described from Florida and Gulf of Mexico, respectively, whereas *B*. *pacifica* was described from Mexican Pacific. According to [21], *B*. *oculata* occurs in the Gulf of Mexico and Caribbean Sea, from Florida to Venezuela; *B*. *pacifica* is found in the eastern tropical Pacific Ocean, while *B*. *lamellifera* occurs in Bermuda, Gulf of Mexico, Venezuela and, probably, also Curaçao.

**Table 3:** Morphological comparison among species of *Branchiosyllis* with well developed branchiae.

	*Branchiosyllis lamellifera*	*Branchiosyllis tamandarensis sp. n.*	*Branchiosyllis oculata*	*Branchiosyllis pacifica*
Original description	Verril 1900	This paper	Ehlers 1887	Rioja 1941
Additional descriptions	[76](as *B*. *oculata*) and [28]		[77] [73]	[78]
Colour pattern	Yellowish, brown, purple, depending upon the sponge in which the specimens dwell	Yellowish to orange	Uniformily dark (brown to black)	Absent
Length (mm)	20	5.5	21	8.5
Width (mm)	2	0.8	2.8	1.5
Number of chaetigers	140	52	112	71
Shape of branchiae	Up to three lobes	Up to six lobes	Dome or slightly flattened	Up to four lobes
Number of anterior/posterior ungulae	3/3	4-6/2-3	3-5/3-5	2-4/2-4
Pharynx lenght (number of segments)	9	5	6	5–7
Proventricle lenght (number of segments)	9	3.5–5	8	4–5
Number of muscle cell rows in proventricle	30	25–30	22	22
Habitat	On and within sponges	On and within sponges	Sand, algae, coral rubble	Coral rubble, algae
Distribution	Bermudas, Gulf of Mexico	Only known from type locality	Caribbean Sea (Florida, Cuba, México, Venezuela)	Eastern Tropical Pacific (Mexico, Panama)

Members of *B*. *oculata* are dark, from brown to black, and the branchiae are dome shaped, different from *B*. *tamandarensis* **sp. n.,** which has yellow to orange body and multi-lobed branchiae. *Branchiosyllis pacifica* has multi-lobed branchiae but differs from *B*. *tamandarensis* **sp. n.** by having shorter median and lateral antennae, with 8–9 and 10 articles each, respectively; shorter dorsal and peristomial cirri, with 16 and 10–12 articles each, respectively; shorter anterior and midbody dorsal cirri, with 14–17 and 13–17 articles each, respectively; longer posterior dorsal cirri, with 10–26 articles each and 2–4 ungulae per anterior parapodium [21]. On the other hand, *B*. *tamandarensis* **sp. n.** has longer appendages (i. e. antennae, peristomial, anterior and midbody dorsal cirri, Table 3) and has 4–6 ungulae on each anterior parapodium.

*Branchiosyllis lamellifera*, according to the redescription by [21], is very similar to *B*. *tamandarensis* **sp. n.**, in overall body shape. However, *B*. *tamandarensis* **sp. n.** has the median antenna inserted more posteriorly in relation to lateral antennae; prostomium slightly broader than long; appendages (i. e. antennae, peristomial and dorsal cirri throughout) more slender and elongated; midbody branchiae with up to six lobes; up to six ungulae on each anterior parapodium; pharynx through ca. 5 chaetigers and proventricle through 3.5–5 chaetigers. On the other hand, *B*. *lamellifera* has all antennae inserted nearly in line (Fig 4B, D in [21]); prostomium conspicuously broader than long; stouter appendages; midbody branchiae with up to three lobes; up to 3–4 ungulae on each anterior parapodium; and pharynx and proventricle both through 9 chaetigers.

#### Biology

Some specimens were found associated with the sponge *Tedania ignis*. However, our methodology does not allow to state that this species lives exclusively within *T*. *ignis*, instead of also being present in other different types of substrates, including other species of sponges, found close to *T*. *ignis*.

Some specimens have one mid-dorsal papilla per chaetiger, arranged in a longitudinal row at dorsal midline (Fig 10C and 10F), while in others such papillae are absent (or at least not visible). We considered this as an intraspecific variation, as it also occurs among members of *B*. *pacifica* (Fig 8A [21]), although this character was not mentioned in [21].

#### Etymology

This species is named after the city of Tamandaré (state of Pernambuco), where Praia dos Carneiros, the type locality, is located. Praia dos Carneiros is one of the most beautiful beaches in the world.

#### Distribution

Atlantic Ocean: Brazil (states of Paraíba and Pernambuco).

Genus *Haplosyllis* Langerhans, 1887

Type species: *Syllis spongicola* Grube, 1855.

#### Diagnosis

Body of variable size, usually up to 10 mm long, with numerous nearly cylindrical segments. Palps robust, fused at bases. Prostomium with four eyes and three antennae. Two pairs of peristomial cirri. Antennae, peristomial, anal and dorsal cirri throughout moniliform, the latter sometimes with a single small article on either side of posterior parapodia. Ventral cirri digitiform. Pharynx with anterior tooth, opening surrounded by crown of 10–12 soft papillae and ring of cilia, occasionally with trepan of small teeth. All chaetae simple, with bi- or unidentate tips, 1–12 per parapodium. Reproduction by acephalous stolon.

#### Remarks

A large revision of this genus was recently carried out, providing descriptions for all known species [19]; also, a complete comparative table with all valid species was recently provided by [10]. The latter authors described two new species from the northeastern Brazilian coast, *H*. *amphimedonicola* Paresque & Nogueira, 2014, living associated with the sponge *Amphimedon viridis* Duchassaing & Michelotti, 1864, and *H*. *rosenalessoae* Paresque & Nogueira, 2014, both from material collected off Paraíba. *Haplosyllis spongicola*, considered for a long time as a cosmopolitan species, was also reported from Pará, northern Brazil, to São Paulo, southeastearn Brazil [30]. However, according to [19] true *H*. *spongicola* is limited to Mediterranean and European Atlantic waters. Since Brazilian reports for this species could not be verified, due to the lack of voucher material, its presence in Brazil is doubtful.

### Identification key to the species of *Haplosyllis* currently recorded in Brazilian waters

1a Two types of chaetae per parapodium along body, different in size and shape. Long chaetae with apical teeth similar in length or distal tooth slightly thinner; short chaetae with larger distal tooth… ***Haplosyllis lattigae* sp. n**
1b Single type of chaetae per parapodium, only different in size… **2**
2a.(1b) Chaetae with denticles from base of proximal tooth to upper side of main fang… ***Haplosyllis loboi***
2b.(1b) Chaetae with denticles only at upper side of main fang… **3**
3a.(2b) Midbody dorsal cirri alternating long, with 3–7 articles, and short, with 1–3 articles each. Body wall of mid- to posterior body chaetigers without dorsal inclusions… ***Haplosyllis amphimedonicola***
2b.(2b) Midbody dorsal cirri alternating long, with 8–13 articles, and short, with 4–8 articles each. Body wall of mid- to posterior body chaetigers with dorsal iridescent and granulose inclusions, arranged in continuous transverse rows across chaetigers, broader laterally, at bases of parapodia, progressively narrowing towards dorsal midline… ***Haplosyllis rosenalessoae***

*Haplosyllis amphimedonicola* Paresque & Nogueira, 2014

*Haplosyllis amphimedonicola* Paresque & Nogueira, 2014: 597–602, Figs 1–4 [10].

#### Material examined

Project *‘BioPol-NE’*. State of Paraíba: Mataraca, Barra de Camaratuba (06º36'S 34º57'W), intertidal: 18 specimens, coll. 12 August 2010. Baía da Traição, Praia do Farol (06º41'S 34º55'W), intertidal: 347 specimens, coll. 09 August 2010. Rio Tinto, Barra de Mamanguape (06º45'S 34º55'W), intertidal: 650 specimens, coll. 11 August 2010. Cabedelo, Píer de Cabedelo (06º58'S 34º50'W), intertidal: 21 specimens, coll. 12 February 2009. Conde, Praia de Tabatinga (07º19'S 34º47'W), intertidal: 361 specimens; coll. 17 September 2012; Praia do Coqueirinho (07º18’S 34º47’W), 294 specimens, coll. 28 August 2011. State of Pernambuco: Goiana, Pontas de Pedra (07°37'S 34°48'W), 318 specimens, coll. 13 December 2012. Ilha de Itamaracá, Ponta do Jaguaribe (07°44'S 34°49'W), intertidal: 61 specimens, coll. 11 December 2012. Recife, Praia de Boa Viagem (08°7.420'S 34°53.725'W), intertidal: 7 specimens, coll. 19 January 2014. Ipojuca, Praia de Muro Alto (08°25.787'S 34°58.567'W), intertidal: 4 specimens, coll. 17 January 2014. Tamandaré, Praia dos Carneiros (08°42.849'S 35°4.999'W), intertidal: 6 specimens, coll. 22 July 2013.

#### Diagnosis

Species of *Haplosyllis* with midbody dorsal cirri alternating long (3–7 articles) and short (1–3 articles), long cirri not reaching half body width at corresponding chaetiger. Chaetae bidentate, often with eroded tips; main fang about same length or shorter than shaft width; mid-joining point straight and long; upper side of main fang with few, short denticles.

#### Remarks

This species was described from northern Paraíba. The distribution range is herein expanded to southern Pernambuco.

#### Type locality

Barra de Mamanguape, Rio Tinto, Paraíba, Brazil (Atlantic Ocean)

#### Distribution

Atlantic Ocean: northeastern Brazil (states of Paraíba and Pernambuco).

*Haplosyllis rosenalessoae* Paresque & Nogueira, 2014

*Haplosyllis rosenalessoae* Paresque & Nogueira, 2014: 603–608, Figs 5–7 [10].

#### Material examined

Project *‘BioPol-NE’*. State of Paraíba: Mataraca, Barra de Camaratuba (06º36'S 34º57'W), intertidal: 4 specimens, coll. 12 August 2010. Baía da Traição, Praia do Farol (06º41'S 34º55'W), intertidal: 32 specimens, coll. 09 August 2010. Rio Tinto, Barra de Mamanguape (06º45'S 34º55'W), intertidal: 9 specimens, coll. 11 August 2010. Cabedelo, Píer de Cabedelo (06º58'S 34º50'W), intertidal: 13 specimens, coll. 12 February 2009. Conde, Praia de Tabatinga (07º19'S 34º47'W), intertidal: 15 specimens; coll. 17 September 2012; Praia do Coqueirinho (07º18’S 34º47’W), 11 specimens, coll. 28 August 2011. State of Pernambuco: Goiana, Pontas de Pedra (07°37'S 34°48'W), 24 specimens, coll. 13 December 2012. Ilha de Itamaracá, Ponta do Jaguaribe (07°44'S 34°49'W), intertidal: 8 specimens, coll. 11 December 2012. Recife, Praia de Boa Viagem (08°7.420'S 34°53.725'W), intertidal: 4 specimens, coll. 19 January 2014.

#### Diagnosis

*Haplosyllis* with dorsal inclusions on midbody chaetigers. Chaetae with main fang shorter than shaft width; apical teeth close to each other, chaetae nearly unidentate at first glance; aciculae distally curved.

#### Remarks

This species was also described from Paraíba. The distribution range is herein expanded to southern Pernambuco.

#### Type locality

Praia de Cabo Branco, João Pessoa, Paraíba, Brazil (Atlantic Ocean)

#### Distribution

Atlantic Ocean: northeastern Brazil (states of Paraíba and Pernambuco).

*Haplosyllis lattigae* sp. n.

**Fig 14:**
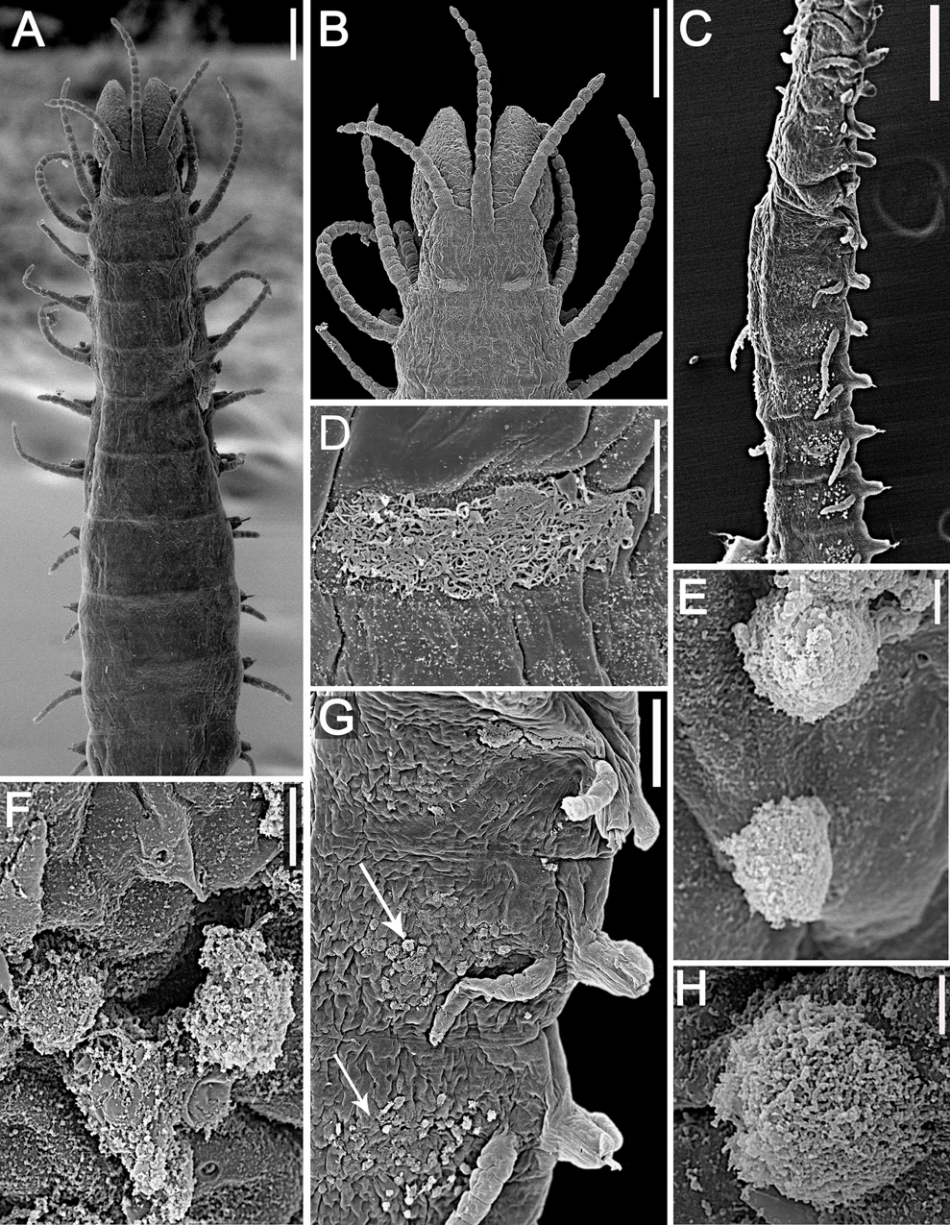
*Haplosyllis lattigae* sp. n., SEM. (A) (B) anterior end, dorsal view; (C) midbody segments, dorso-lateral view; (D) detail of nuchal organ; (E) (F) details of dorsal papillae; (G) midbody parapodia, dorso-lateral view, arrows indicate dorso-lateral papillae; (H) detail of dorsal papilla. Scale bars: A, C, 200 μm; B, 100 μm; D, 10 μm; E, H, 2 μm; F, 5 μm; G, 50 μm.

**Fig 15:**
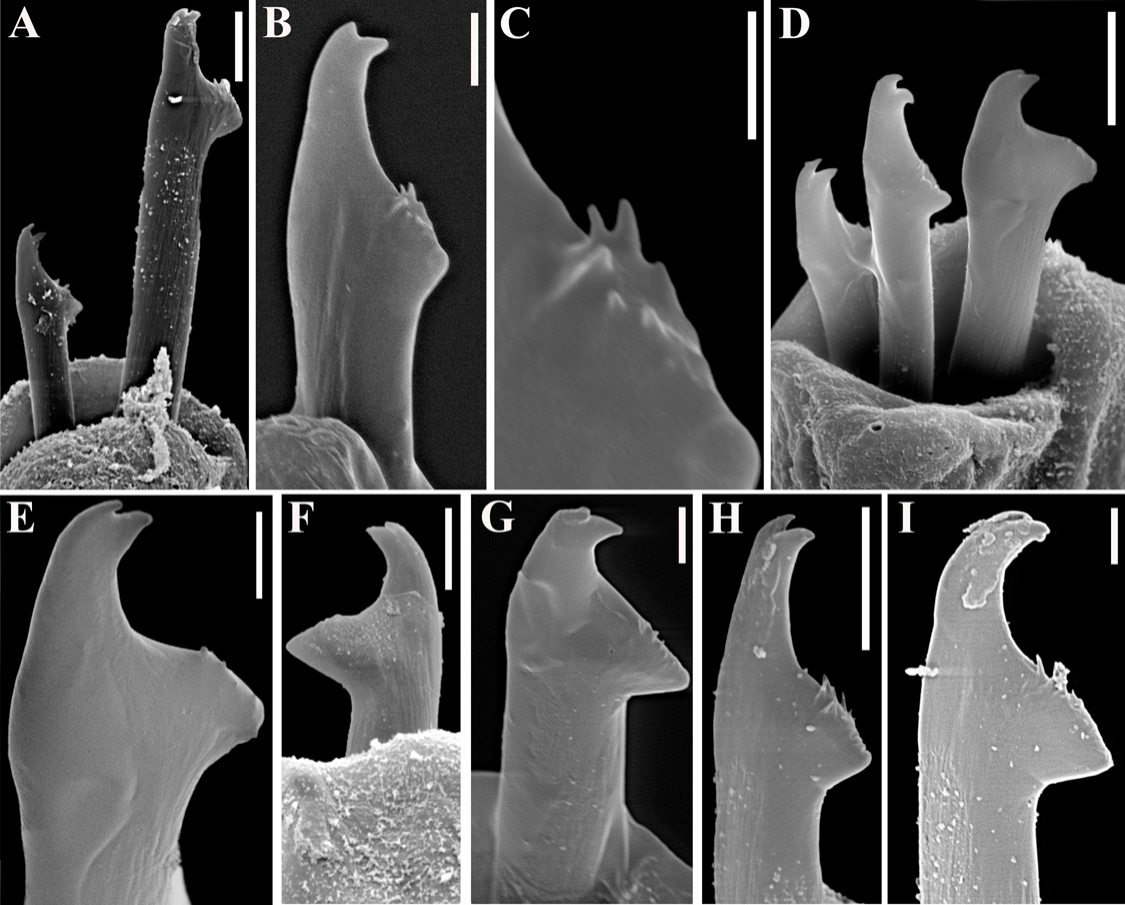
*Haplosyllis lattigae* sp. n. (A) chaetae, parapodium 5; (B) chaeta, parapodium 6; (C) detail of chaeta, parapodium 6; (D) chaetae, parapodium 11; (E) longer chaeta, parapodium 11; (F) shorter chaeta, midbody parapodium; (G) longer chaeta, posterior parapodium; (H) (I) shorter chaetae, posterior parapodia. Scale bars: A, D, F, H, 5 μm; B, E, G, I, 2 μm; C, 1 μm.

**Fig 16:**
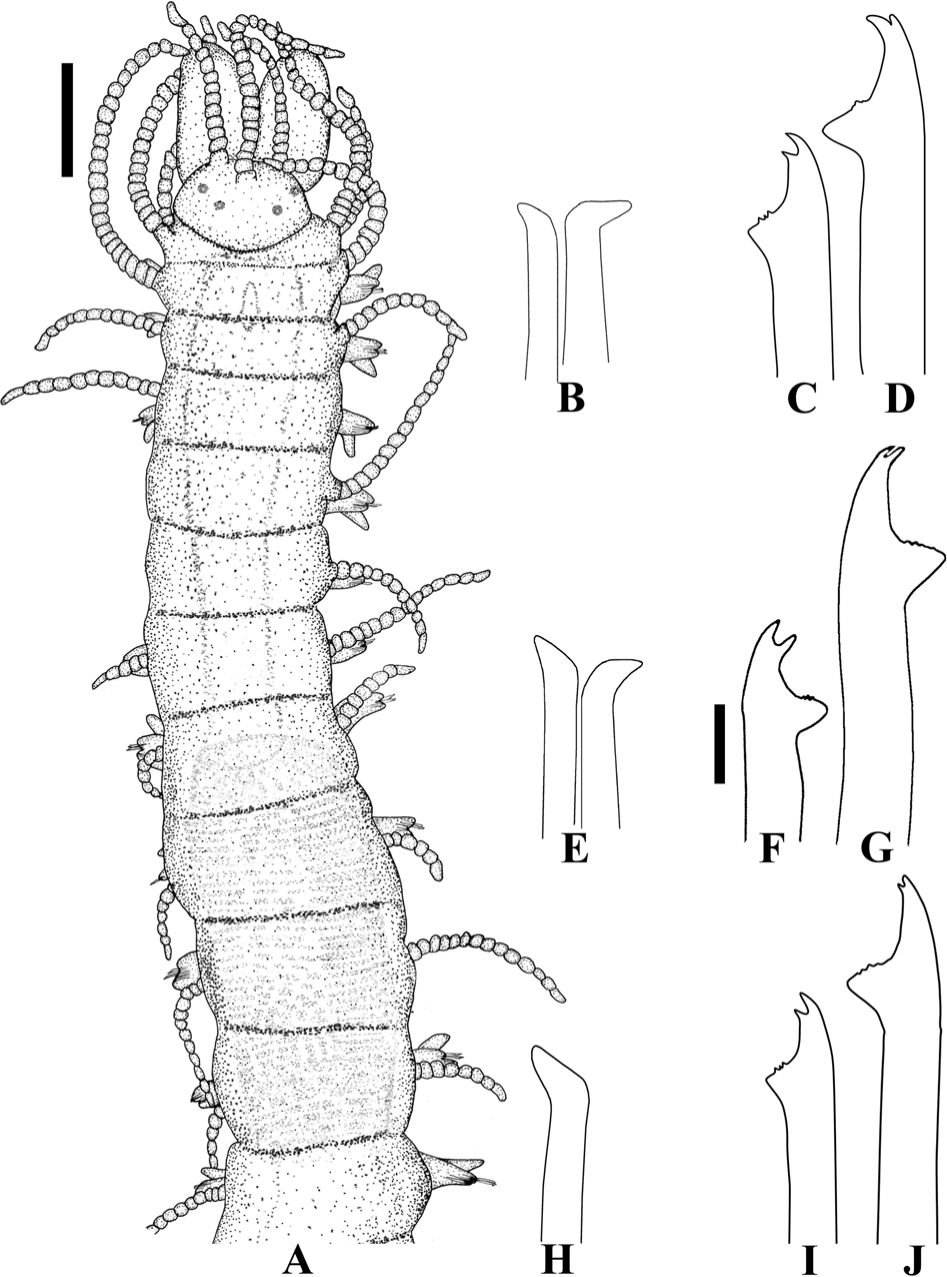
*Haplosyllis lattigae* sp. n. (A) anterior body, dorsal view; (B) aciculae, anterior parapodium; (C) (D) short and long chaetae, respectively, anterior body; (E) aciculae, midbody; (F) (G) short and long chaetae, respectively, midbody; (H) acicula, posterior body; (I) (J) short and long chaetae, respectively, posterior body. Scale bars: A, 200 μm; B–J, 5 μm.

Figs 14–16; Tables 4 and 5

urn:lsid:zoobank.org:act:65728345-F46A-4EBE-B416-7B31B46B3F81

**Table 4:** Morphological variation among the type series of *Haplosyllis lattigae* sp. n. (inc. = incomplete;? = missing data).

	MZUSP 2861	MZUSP 2862	MZUSP 2863	MNCN 16.01/16879	MNCN 16.01/16879
	Holotype	Paratype 1	Paratype 2	Paratype 3	Paratype 4
Body length (mm)	6.5	5	5.5	4	0.35
Body length until chaetiger 10 (mm)	2	1.5	1.9	1.2	1.1
Body width (mm)	0.5	0.5	0.4	0.3	0.35
Number of chaetigers	36	37	41	35	34
**Proventricle**					
Length (mm)	0.7	1	0.7	0.5	0.8
Number of chaetigers	3	5	5	3	3
Number of rows of muscle-cells	39	36	29	41	37
Pharynx length (number of segments)	6	3	5		3 (ev.)
**Number of articles**					
Median antennae	22	13	19	18	21
Lateral antennae	14, 15	11, 12	16, 17	10, 10	13, 2 (inc.)
Dorsal peristomial cirri	18, 19	16, 16	19, 21	14, 15	16, 18
Ventral peristomial cirri	9, 9	9, 10	8, 9	7, 8	9,
Dorsal cirri 1	23, 19	14,17	23, 23	17, 18	20, 22
Dorsal cirri 2	6, 9	8, 6	10, 9	7, 7	8, 9
Dorsal cirri 3	12, 10	7 (inc,), 9	14, 13	7, 8	11, 12
Dorsal cirri 4	13, 11	10, 11	14, 14	10, 10	12, 13
Dorsal cirri 5	7, 5	5, 6	9, 8	6, 6	8, 8
Dorsal cirri 6	7, 7	9, 10	13, 14	7, 8	12, 10
Dorsal cirri 7	8,?	3, 4	7, 5	5, 5	6, 6
Dorsal cirri 8	7,6	4, 4	5, 8	5, 5	6, 6
Long cirri, midbody	6–11	6–7	7–9	6–8	6–7
Short cirri, midbody	4–6	3–4	4–6	3–4	4–5
Anal cirri	9, 10	6, 8	7, 8	6, 8	6, 6

**Table 5:** Comparison between *H*. *lattigae* sp. n. and the most similar congener, *H*. *djiboutiensis*, from different localities around the world.

	*H. djiboutiensis*	*H. djiboutiensis*	*H. djiboutiensis*	*H. djiboutiensis*	*H. lattigae sp. n.*
Source	[19]	[31]	[31]	[32]	Present paper
Locality	Djubal (Polytype MNHN 48)	Qatar	Abu Dhabi	Australia (Western Australia and New South Wales)	Brazil
Total length x width at proventricle level (mm)/ number of chaetigers	8 x 0.35/38	2–4 x? / 21–42	3–9 x? / 25–57	3–17 x? / 30–92	4–6.5 x 0.3–0.5 / 34–41
**Proventricle**					
Length (mm), chaetigers/number of muscle-cells rows	0.45, 4/36	?, 5–12 / 27–36	?, 4–6 / 37–41	?, 3-9/30-50	0.5–1.0, 3–4.5 / 30–40
Pharynx length (number of segments)	5	?	?	4–6	3–6
**Number of articles**					
Median, lateral antennae	22,14	10–25, 7–18	12–21, 8–29	11–30,7–16	13–22, 10–17
Dorsal, ventral peristomial cirri	13–15,7–11	7–18,?	10–40,?	13–24,?	14–21,7–10
Dorsal cirri segs 1–6	23, 12, 15, 19, 10, 15	11–25, 2–13, 4–15, 5–16, 2–9, 3–13	10–44, 4–21, 5–30, 8–27, 4–23, 5–24	11–30, 5–16, 7–20, 10–29, 4–16, 6–24	19–23, 6–10, 7–12, 10–14, 5–9, 7–14
Midbody long, short dorsal cirri	7–9, 4–5	?	?	7–16, 3–11	6–11, 3–6
Anal cirri	?	6–7	1–11	8–10	6–10
**Aciculae**					
Number per parapodium (anterior, midbody, posterior body)	2,2,2	?	?	2,2,1	2,2,1
Morphology	1–2 curved tips directed upwards	?	?	Anterior and midbody: 1- straight, 2- curved tip directed upwards; Posterior: 1- curved tip directed upwards	Anterior and midbody: 1- subdistally curved at almost right angle and oblique tip, 2- distally sinous, directed upwards; posterior: 1- same as type 2
**Chaetae in anterior parapodia**					
Differences between chaetae on same parapodium	No	No	No	No	In size and shape
Size of main fang (compared to shaft width)	Conpicuously shorter	Conpicuously shorter	Conpicuously shorter	Conpicuously shorter	Shorter
Main fang upper side	With denticles	With denticles	With denticles	With denticles	With denticles
Mid-joining point	Straight and long	Straight and long	Straight and long	Straight and long	Large chaetae: straight and long; short chaetae: short
Apical teeth	Similar in length	Similar in length	Similar in length	Similar in length	Large chaetae: similar in length or distal tooth slightly thinner; short chaetae: distal tooth larger than proximal one, teeth directed forwards

#### Material examined

Project ‘*BioPol-NE*’. State of Paraíba: Mataraca, Barra de Camaratuba (06º36'S 34º57'W), intertidal: 2 specimens, coll. 12 August 2010. Baía da Traição, Praia do Farol (06º41'S 34º55'W), intertidal: 3 specimens, coll. 09 August 2010. Rio Tinto, Barra de Mamanguape (06º45'S 34º55'W), intertidal: 6 specimens, coll. 11 August 2010. Cabedelo, Píer de Cabedelo (06º58'S 34º50'W), intertidal: 2 specimens, coll. 12 February 2009. State of Pernambuco: Goiana, Pontas de Pedra (07°37'S 34°48'W), 4 specimens, coll. 13 December 2012. Ilha de Itamaracá, Ponta do Jaguaribe (07°44'S 34°49'W), intertidal: 2 specimens, coll. 11 December 2012. Sirinhaém, Barra do Sirinhaém (08°36.707'S 35°2.450'W), intertidal: 1 specimen, coll. 23 July 2013. São José da Coroa Grande, recifes São José da Coroa Grande (08°53.779'S 35°8.239'W), intertidal: 1 specimen, coll. 25 June 2013. Tamandaré, Praia dos Carneiros (08°42.849'S 35°4.999'W), intertidal: 3 specimens, coll. 22 July 2013.

#### Type series

All specimens collected by the Project ‘*BioPol-NE*’. State of Paraíba: Baía da Traição, Praia do Farol (06°41.331'S 34°55.803'W), intertidal: Holotype (MZUSP 2861); Paratypes 1–2 (MZUSP2832-2863); Paratypes 3–4 (MNCN 16.01/16879), coll. 09 August 2010. Morphological data of specimens of the type series is provided in Table 4.

#### Additional material examined

*Haplosyllis carmenbritoae* Lattig, San Martín & Martin, 2007: Holotype (MNCN 16.01/10645), Tabaiba, Santa Cruz de Tenerife, Canary Islands, Spain, 100 m deep, coll. 1990; 6 paratypes (MNCN 16.01/10646), Tabaiba, Santa Cruz de Tenerife, Canary Islands, Spain, 100 m deep, coll. 1990. *Haplosyllis granulosa* (Lattig, Martin & San Martín, 2007): Holotype (MNCN 16.01/10606), Nerja, Málaga, Andalucía, Spain, 3–4.5 m deep, coll. 24 February1983 by G. San Martín; 5 paratypes (MNCN 16.01/10607), Nerja, Málaga, Andalucía, Spain, 3–4.5 m deep, coll. 24 February1983 by G. San Martín.

#### Description

Small-sized body, largest specimen analysed ca. 6.5 mm long, 0.5 mm wide, with 36 chaetigers (Table 4). Pigmentation absent, specimens beige to light yellow after preservation. Mid- to posterior body chaetigers with small dorso-lateral papillae close to dorsal cirri (Fig 14E–14H). Triangular, distally rounded palps, totally free from each other (Fig 14B). Sub-pentagonal prostomium, shorter than palps, with two pairs of red eyes in trapezoidal arrangement, anterior eyespots absent; median antenna inserted between anterior eyes, longer than palps (Figs 14A and 14B, 16A), with 13–22 articles; lateral antennae shorter, inserted at anterior margin of prostomium, with 10–18 articles each (Figs 14A and 14B; 16A). Ciliated nuchal organs between prostomium and peristomium (Fig 14A and 14B and 14D), only visible under SEM. Peristomium of similar length or slightly shorter than following chaetigers; dorsal peristomial cirri shorter than median antenna, with 14–21 articles each; ventral peristomial cirri shorter, with 7–11 articles each. Anterior dorsal cirri relatively long (Figs 14A and 14B, 16A); dorsal cirri of chaetiger 1 longer than following cirri, with 14–23 articles each, reaching slightly beyond tip of palps; dorsal cirri of chaetiger 2 shorter, with 6–10 articles each; dorsal cirri of chaetiger 3 with 7–14 articles each; dorsal cirri of chaetiger 4 longer, with 10–14 articles each; dorsal cirri of chaetigers 5 and 6 with 5–9 and 6–14 articles each, respectively; dorsal cirri on chaetigers 7 and 8 similar to each other, shorter, with 5–9 articles each; following cirri alternating long, with 6–11 articles, and short, with 3–6 articles each, long cirri about half body width at corresponding segment (Fig 14C); alternation between long and short cirri progressively less conspicuous posteriorwards; posterior dorsal cirri short, with 1–3 articles each. Ventral cirri ovate, inserted at bases of parapodia (Fig 14G), slightly longer than parapodial lobes on anterior body, shorter on posterior body. Parapodial lobes conical (Fig 14G), with 1–3 chaetae per parapodium. Anterior parapodia with two types of chaetae each, if more than 1 chaeta are present, long chaetae (Figs 15A–15C; 16D) with apical teeth of same size or distal tooth slightly thinner, teeth with oblique tip, upwardly-directed; main fang shorter than shaft width; mid-joining point straight and relatively long; upper side of main fang with few, short denticles; short chaetae (Figs 15A; 16C) with apical teeth forward directed, distal tooth larger, with relatively wide, nearly rounded space between teeth; main fang shorter than shaft width; mid-joining point straight and relatively shorter; upper side of main fang with few, short denticles. From midbody parapodia, long chaetae (Figs 15D–15G; 16G and 16J) with apical teeth of different size, subdistal tooth larger, with narrow space in between, teeth with oblique tip; main fang shorter than shaft width; mid-joining point straight and relatively short; upper side of main fang smooth; short chaetae (Figs 15D, 15H and 15I; 16F and 16I) with apical teeth larger, with wider space in between, teeth with oblique tip; main fang shorter than shaft width; mid-joining point curved and relatively long; upper side of main fang with short denticles. Anterior and midbody chaetigers with 2 aciculae per parapodium, single acicula per parapodium on posterior body; aciculae of two types, one of which subdistally bent at right angle, with oblique tip, another distally oblique, upwardly directed (Fig 16B and 16E); posterior body parapodia with latter type of acicula only (Fig 16H). Pygidium semicircular, with ciliated anus dorsally and pair of anal cirri with 6–10 articles each, up to three times as long as posterior dorsal cirri, distal articles elongated. Pharynx extending for 3–6 chaetigers, tooth at anterior border; anterior margin of pharynx surrounded by fringe of cilia; proventricle 0.7–1 mm long, extending for 3–5 chaetigers, with 29–41 rows of muscle cells.

#### Remarks

The most similar species to *Haplosyllis lattigae ***sp. n**. is *H*. *djiboutiensis* Gravier, 1900, which is recognized by the alternation in length of dorsal cirri on midbody, abundant dorsal granules on posterior body, anterior chaetae with very short main fang, with short denticles on upper side, and presence of two types of chaetae per parapodium from midbody chaetigers, short chaetae with short proximal tooth, large chaetae with mid-joining point long and straight, and apical teeth equal in size [31]. Material of *H*. *djiboutiensis* from Australia [32], Djubal (Qatar) and Abu Dhabi, including paratypes [31], was recently analysed; however, although slight morphological differences were noticed among populations, those are too slight characterize different species (Table 5).

*Haplosyllis lattigae* **sp. n.** differs from *H*. *djiboutiensis* by having two types of chaetae of different sizes also on anterior parapodia, long chaetae with apical teeth of similar size or distal tooth slightly thinner, mid-joining point straight and relatively long, main fang proportionally longer when compared with that of *H*. *djiboutensis*; and short chaetae with apical teeth forward directed, distal tooth larger than proximal one, with relatively wide, nearly rounded space between teeth, and mid-joining point straight and relatively short. Additionally, *H*. *lattigae* **sp. n.** differs from *H*. *djiboutiensis* from Djibuti [19] by having shorter appendages anteriorly (Table 5).

*Haplosyllis lattigae* **sp. n.** differs from *H*. *amphimedonicola* and *H*. *rosenalessoae* mainly by having two types of chaetae per parapodium along the body. Furthermore, in contraposition to *H*. *lattigae* **sp. n.**, *H*. *amphimedonicola* has a stouter body; papillae absent on dorsum; shorter antennae and midbody cirri; chaetae with distal teeth similar in size to each other; and aciculae with different morphology. Compared to *H*. *lattigae* **sp. n.**, *H*. *rosenalessoae* is a longer and stouter species; with shorter and stouter antennae; chaetae on mid- and posterior body with smooth main fang; distal teeth inconspicuous from midbody; and aciculae also with different morphology.

#### Type locality

Praia do Farol, Baía da Traição, Paraíba, Brazil (Atlantic Ocean).

#### Distribution

Atlantic Ocean: Brazil (states of Paraíba and Pernambuco).

#### Etymology

This species is named in honor of Dr. Patricia Lattig from MNCN for her great contributions to the taxonomy of *Haplosyllis* in the last years.

*Haplosyllis loboi* Paola, San Martín & Martin, 2006

Figs 17–19

*Haplosyllis loboi* Paola, San Martín & Martin, 2006: 347–352, figs. 1–4 [33]; Lattig & Martin, 2009: 23 [19].

**Fig 17:**
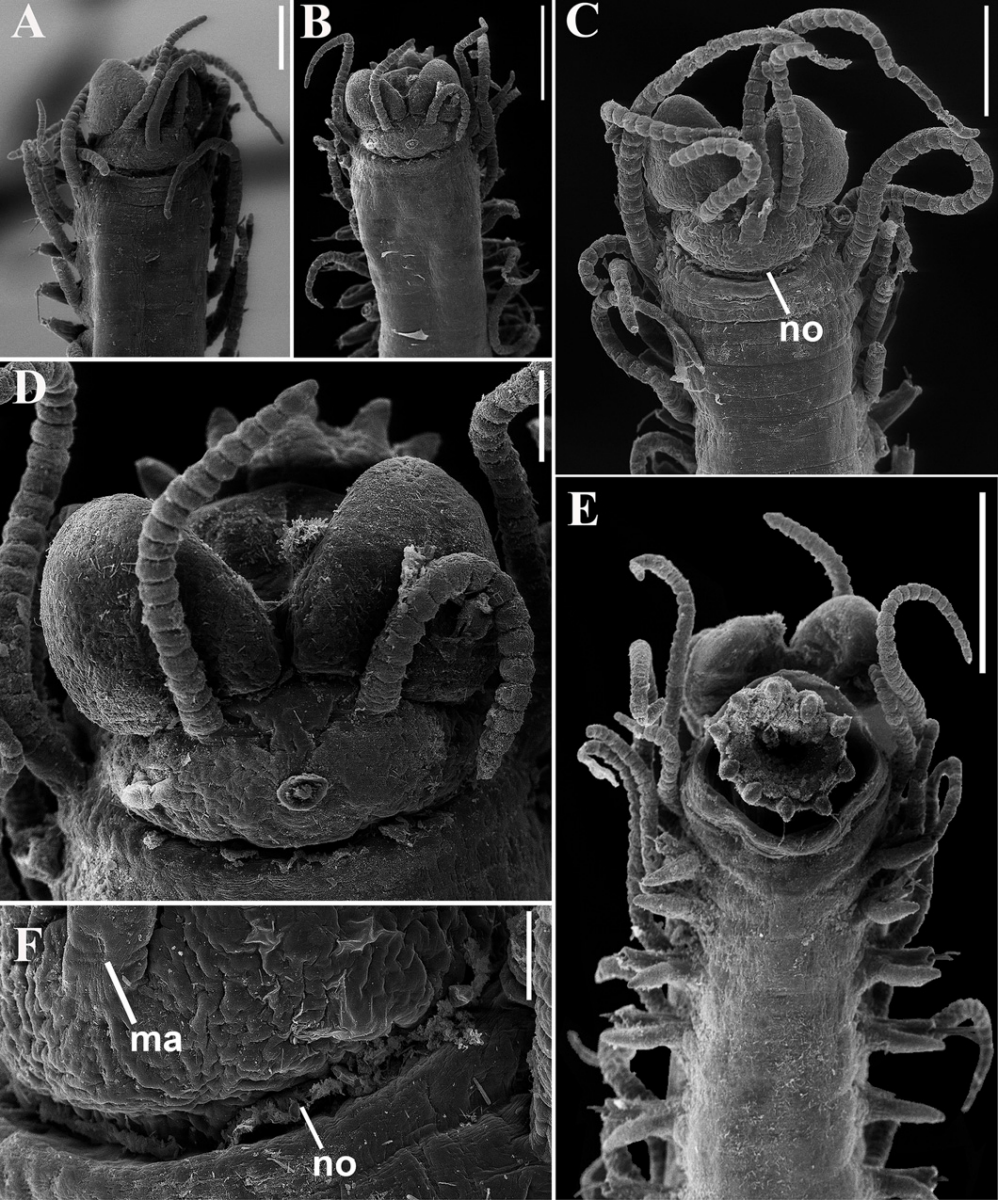
*Haplosyllis loboi*, SEM. (A) (B) (C) anterior end, dorsal view; (D) prostomium and peristomium, dorsal view; (E) anterior end, ventral view; (F) detail of nuchal organ, between prostomium and peristomium, right-hand dorso-lateral view. **ma**—median antenna; **no**—nuchal organ. Scale bars: A, C, 100 μm; B, E, 200 μm; D, 50 μm; F, 20 μm.

**Fig 18:**
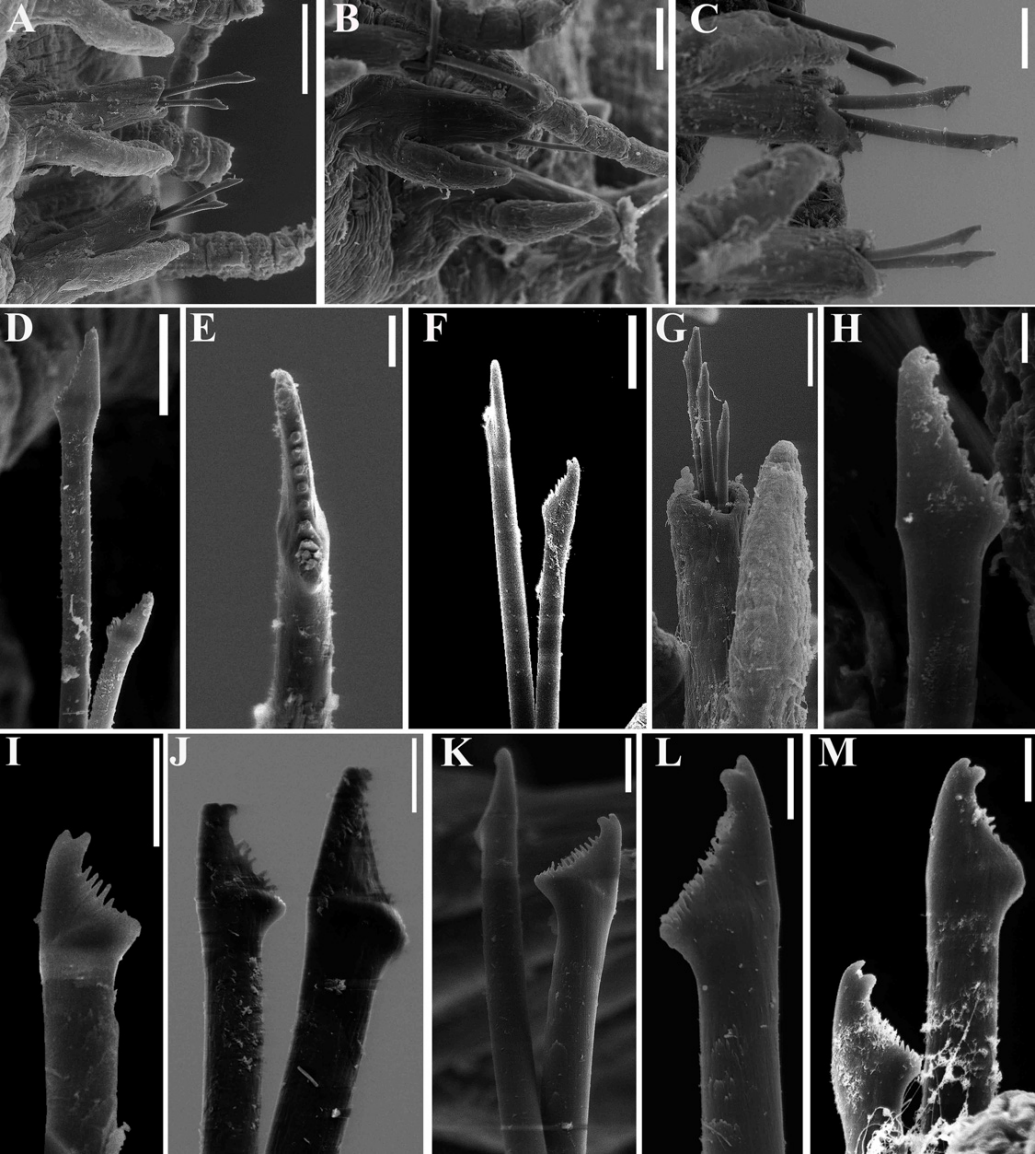
*Haplosyllis loboi*, SEM. (A) anterior parapodia, ventral view; (B) (C) midbody parapodia, ventral views; (D) (E) (F) chaetae, parapodium 2; (G) chaetae, parapodium 5; (H) (I) (J) (K) chaetae, midbody parapodia; (L) (M) chaetae, posterior parapodia. Scale bars: A, 50 μm; B, C, G, 20 μm; D, F, 10 μm; E, H, M, 2 μm; I–L, 5 μm.

**Fig 19:**
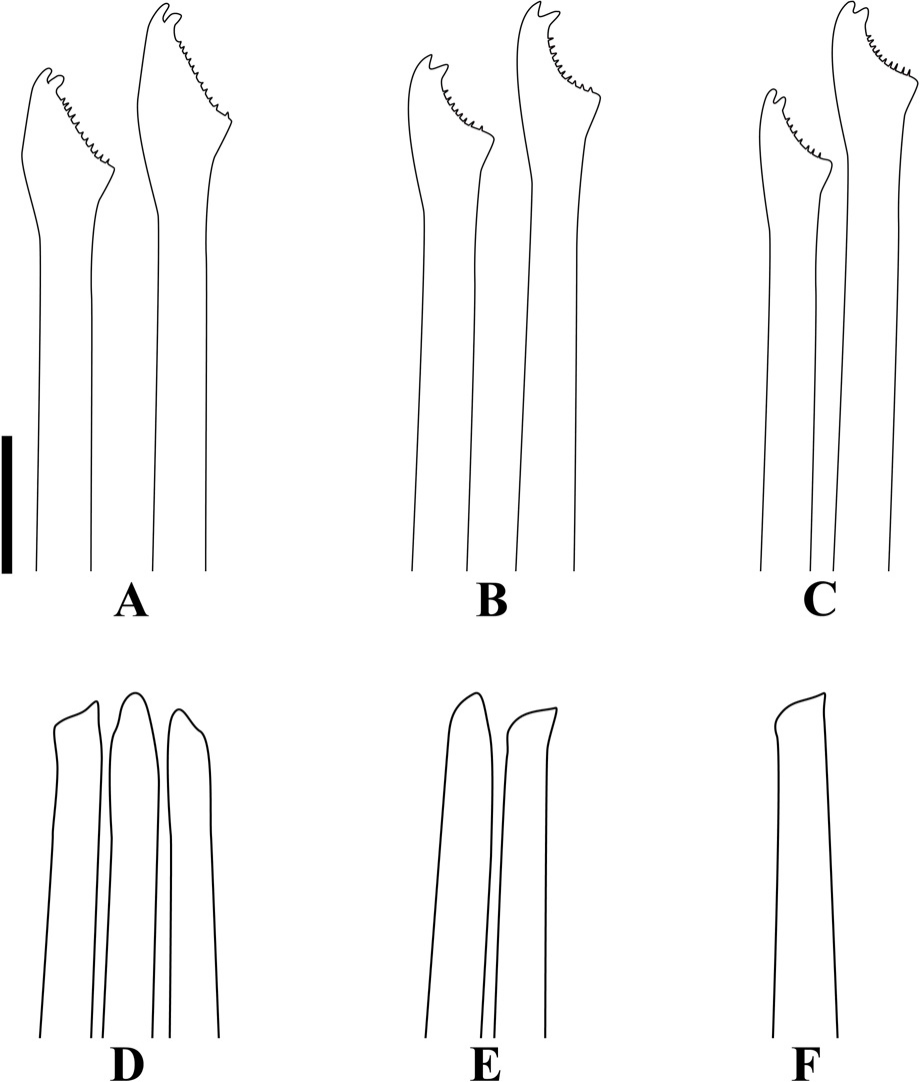
***Haplosyllis loboi***. (A) (B) (C) chaetae, anterior, midbody and posterior parapodia, respectively; (D) (E) (F) aciculae, anterior, midbody and posterior parapodia, respectively. Scale bars: A–F: 10 μm.

#### Material examined

Project ‘*HABITATS*’. State of Rio de Janeiro, Bacia de Campos, soft bottom, van Veen grab: 21°22'58.704''S 40°19'44.429''W, 53 m: 5 specimens (MZUSP 2864), coll. 21 July 2009; 21°42'37.461''S 40°8'59.105''W, 147 m: 13 specimens (MZUSP 2865), coll. 9 March 2009; 21°42'53.677''S 40°10'16.159''W, 99 m: 6 specimens (MZUSP 2866), coll. 9 March 2009; 21°42'53.795''S 40°10'15.120''W, 98 m: 6 specimens (MZUSP 2867), coll. 7 July 2009; 21°43'10.796''S 40°11'30.783''W, 71 m: 16 specimens (MZUSP 2868), coll. 7 July 2009; 21°43'9.996''S 40°11'30.593''W, 73 m: 23 specimens (MZUSP 2869), coll. 9 March 2009; 22°19'32.030''S 40°37'8.991''W, 75 m: 1 specimen (MZUSP 2870), coll. 15 March 2009; 22°23'39.043''S 40°20'41.990''W, 149 m: 1 specimen (MZUSP 2871), coll. 4 July 2009; 22°23'39.180''S 40°20'41.201''W, 152 m: 8 specimens (MZUSP 2872), coll. 23 February 2009; 22°31'7.299''S 40°31'32.527''W, 139 m: 7 specimens (MZUSP 2873), coll. 22 February 2009; 22°31'7.826''S 40°31'32.004''W, 138 m: 2 specimens (MZUSP 2874), coll. 3 July 2009; 22°3'39.049''S 40°6'59.816''W, 90 m: 6 specimens (MZUSP 2875), coll. 6 July 2009; 22°3'45.381''S 40°9'59.186''W, 76 m: 21 specimens (MZUSP 2876), coll. 6 July 2009; 22°3'45.625''S 40°9'59.188''W, 75 m: 5 specimens (MZUSP 2877), coll. 25 February 2009; 22°4'14.420''S 40°6'59.597''W, 91 m: 2 specimens (MZUSP 2878), coll. 25 February 2009; 22°46'54.279''S 41°3'33.082''W, 78 m: 2 specimens (MZUSP 2879), coll. 2 July 2009; 22°46'55.270''S 41°3'33.162''W, 78 m: 9 specimens (MZUSP 2880), coll. 22 February 2009; 22°52'1.951''S 40°57'28.983''W, 92 m: 1 specimen (MZUSP 2881), coll. 22 February 2009; 22°52'2.059''S 40°57'29.001''W, 91 m: 2 specimens (MZUSP 2882), coll. 3 July 2009; 22°57'28.366''S 40°50'30.283''W, 142 m: 1 specimen (MZUSP 2883), coll. 21 February 2009; 22°57'28.411''S 40°50'30.517''W, 143 m: 5 specimens (MZUSP 2884), coll. 3 July 2009; 22°57'28.411''S 40°50'30.517''W, 143 m: 4 specimens (MZUSP 2885), coll. 3 July 2009; 22°59'47.541''S 41°21'7.773''W, 77 m: 9 specimens (MZUSP 2886), coll. 2 July 2009; 22°6'10.579''S 40°3'6.634''W, 149 m: 1 specimen (MZUSP 2887), coll. 7 July 2009; 22°6'10.670''S 40°3'6.481''W, 153 m: 6 specimens (MZUSP 2888), coll. 24 February 2009; 23°10'4.780''S 41°3'6.553''W, 107 m: 51 specimens (MZUSP 2889), coll. 21 February 2009; 23°10'5.207''S 41°3'6.453''W, 107 m: 51 specimens (MZUSP 2890), coll. 2 July 2009; 23°11'30.269''S 41°0'47.888''W, 150 m: 34 specimens (MZUSP 2891), coll. 21 February 2009; 23°12'8.577"S 40°59'35.662"W, 141 m: 33 specimens (MZUSP 2892), coll. 2 July 2009; 23°36'14.903"S 41°21'29.953"W, 143 m: 2 specimens (MZUSP 2893), coll. 1 March 2009; 23°36'14.920''S 41°21'30.085''W, 144 m: 12 specimens (MZUSP 2894), coll. 1 July 2009. Project ‘*REVIZEE*’: State of Rio de Janeiro: 23°10'S 40°56'W, 425 m: 3 specimens (MZUSP 2895), coll. 1 March 1998; 24°17'S 44°12'W, 163 m: 2 specimens (MZUSP 2896), coll. 10 January 1998; 25°37'S 45°14'W, 153 m: 6 specimens (3 mounted for SEM), coll. 13 January 1998.

#### Additional material examined

*Haplosyllis loboi*. Argentina, La Plata (38°05'03''S 57°22'W), coarse sand: paratype (MNCN 16.01/9034), coll. 1975 by L. Orensanz, det. G. San Martín.

*Haplosyllis spongiphila* (Verrill, 1885). USA, Massachusetts, off Martha's Vineyard: 10 co-types (USNM 9864), coll. 4 September 1880, A.E. Verrill.

#### Description

Mid-sized body, largest specimen analysed ca. 13 mm long, 0.6 mm wide, with 69 chaetigers. Pigmentation absent. Triangular, distally rounded palps, basally joined by thinner membrane (Fig 17A–17E). Prostomium ovate, shorter than palps (Fig 17A–17D), with two pairs of red eyes in trapezoidal arrangement and pair of eyespots on anterior margin; median antenna inserted between posterior eyes, longer than palps (Fig 17A–17D), with 28–36 articles; lateral antennae shorter, inserted on anterior margin of prostomium, with 19–23 articles each (Fig 17A–17D). Ciliated nuchal organs between prostomium and peristomium (Fig 17F), only visible under SEM. Peristomium shorter than following chaetigers (Fig 17A–17D and 17F); dorsal peristomial cirri shorter than median antenna, with 21–32 articles each; ventral peristomial cirri shorter, with 13–19 articles each. Anterior dorsal cirri relatively longer than remaining ones; dorsal cirri of chaetiger 1 longer than following cirri, with 32–47 articles each, reaching beyond tips of palps (Fig 17C and 17E); dorsal cirri of chaetiger 2 with 17–26 articles each; dorsal cirri of chaetigers 3 and 4 similar to each other, with 27–33 articles each; dorsal cirri of chaetiger 5 with 18–29 articles each; dorsal cirri of chaetiger 6 longer, with 33–35 articles each; dorsal cirri of chaetigers 7 and 8 similar to each other, with 22–26 articles each; following cirri alternating long, with 16–25 articles, and short, with 8–12 articles each, long cirri about twice as long as short cirri, of similar length or slightly longer than body width at corresponding segment; alternation between long and short cirri progressively less conspicuous posteriorwards; posterior dorsal cirri short, with 3–6 articles each, shorter than body width, with elongate articles. Ventral cirri digitiform, inserted at bases of parapodia, slightly longer than parapodial lobes on anterior body (Fig 17E), about same length as parapodial lobes from midbody. Parapodial lobes bilobed (Fig 18A–18C); anterior parapodia with 2–3 bidentate chaetae each; mid- and posterior body parapodia with 2 chaetae each. Chaetae from anterior body chaetigers with main fang shorter than chaetal width; diagonal mid-joining point, with denticles, continuous with denticles on upper side of main fang; apical teeth similar in length, upwardly-directed, with rounded tips, separated by narrow angle, teeth sometimes eroded (Figs 18D–18G; 19A). Chaetae of mid- and posterior body chaetigers with main fang shorter than chaetal width; mid-joining point curved, with denticles, continuous with denticles on upper side of main fang; apical teeth with rounded tips, distal tooth upwardly-directed, thinner, proximal tooth more oblique, and conspicuous space between teeth (Figs 18H–18M; 19B, 19C). Anterior body with 3 aciculae per parapodium, midbody with 2–3 aciculae per parapodium, single acicula per parapodium on posterior body; aciculae straight, with oblique tips (Fig 19D–19F). Pygidium semicircular, with ciliated anus dorsally and pair of anal cirri with 12–16 articles each, twice as long as posterior body width. Pharynx extending for 8–9 chaetigers, tooth at anterior border; anterior margin of pharynx surrounded by 10 papillae and fringe of cilia; proventricle 1.1–1.4 mm long, extending for 5–9 chaetigers, with 47–51 rows of muscle cells.

#### Remarks

*Haplosyllis loboi* has a peculiar chaetal morphology, similar to *Trypanoseta* sp. from Japan [34], with denticles from the base of the proximal tooth to the upper side of the main fang. The genus *Trypanoseta* was synonymized with *Haplosyllis* by [19]. The specimen from Japan differ from those from *H*. *loboi* by having up to 7 chaetae per parapodium, each with two groups of spines, the first in the mid-joining point and the second in the surface of the main fang, with one longer spine in between groups.

Brazilian specimens of *H*. *loboi* differ slightly from the specimens from the type locality (La Plata, Argentina). Although the original description states that there are 2 chaetae per parapodium throughout, we observed some chaetigers with 3 chaetae on some anteriormost parapodia in the paratype examined at the MNCN. In general, dorsal cirri from chaetiger 2 onwards are longer in Brazilian specimens; also, Brazilian specimens have a larger number of muscle-cells rows in proventricle than Argentinean ones (up to 51 x ~36). In addition, chaetal apical teeth seem to be sharper in Argentinean specimens (Fig 4E–4H in [33]) and the denticulation between the base of apical teeth and the surface of main fang is less conspicuous in Brazilian specimens.

#### Biology

*Haplosyllis loboi* was described from coarse sandy sediments, from La Plata, Argentina. The original description suggests an association with sponges, since some specimens were found with several spicules either buried into or attached to the body surface [33]; this was also found in the specimens herein analysed, corroborating the idea of such association.

#### Type locality

La Plata, Argentina (Atlantic Ocean).

#### Distribution

Atlantic Ocean: from northern Rio de Janeiro, Brazil, to La Plata, Argentina.

Genus *Opisthosyllis* Langerhans, 1879

Type species: *Opisthosyllis brunnea* Langerhans, 1879.

#### Diagnosis

Relatively medium to large-sized body. Palps only basally fused. Prostomium with three antennae, two pairs of eyes and sometimes one pair of anterior eyespots. Two pairs of peristomial cirri; peristomium sometimes with occipital flap. Antennae, peristomial and dorsal cirri throughout distinctly articulated; ventral cirri digitiform to ovate. Compound chaetae as falcigers only. Pharynx with opening surrounded by crown of papillae, pharyngeal tooth located away from anterior border, usually on posterior half of pharynx. Reproduction by means of stolons [32].

#### Remarks

The genus was first recorded for the Brazilian coast by [35], which reported *O*. *corallicola* Hartmann-Schröder, 1965 associated with the bryozoan *Schizoporella unicornis* (Johnston in Wood, 1844) off the state of São Paulo. Later on, [12] and [8] recorded *O*. *brunnea* from off the State of São Paulo, and [15] and [8] found *O*. *viridis* Langerhans, 1879 among material from off the states of Espírito Santo and Rio de Janeiro, respectively. The present paper is the first formal record for *Opisthosyllis* for the northeastern Brazilian coast.

### Identification key to the species of *Opisthosyllis* currently recorded in Brazilian waters

1a Dorsum with numerous papillae. Occipital flap absent… ***Opistosyllis viridis***
1b Dorsum without papillae. Occipital flap present… 2
2a.(1a) Ocipital flap long, reaching posterior pair of eyes; anterior compound chaetae subbidentate… ***O*. *brunnea***
2b.(1a) Ocipital flap short, do not reaching posterior pair of eyes; anterior compound chaetae unidentate… ***O*. *corallicola***

*Opisthosyllis brunnea* Langerhans, 1879

Figs 20 and 21

**Fig 20:**
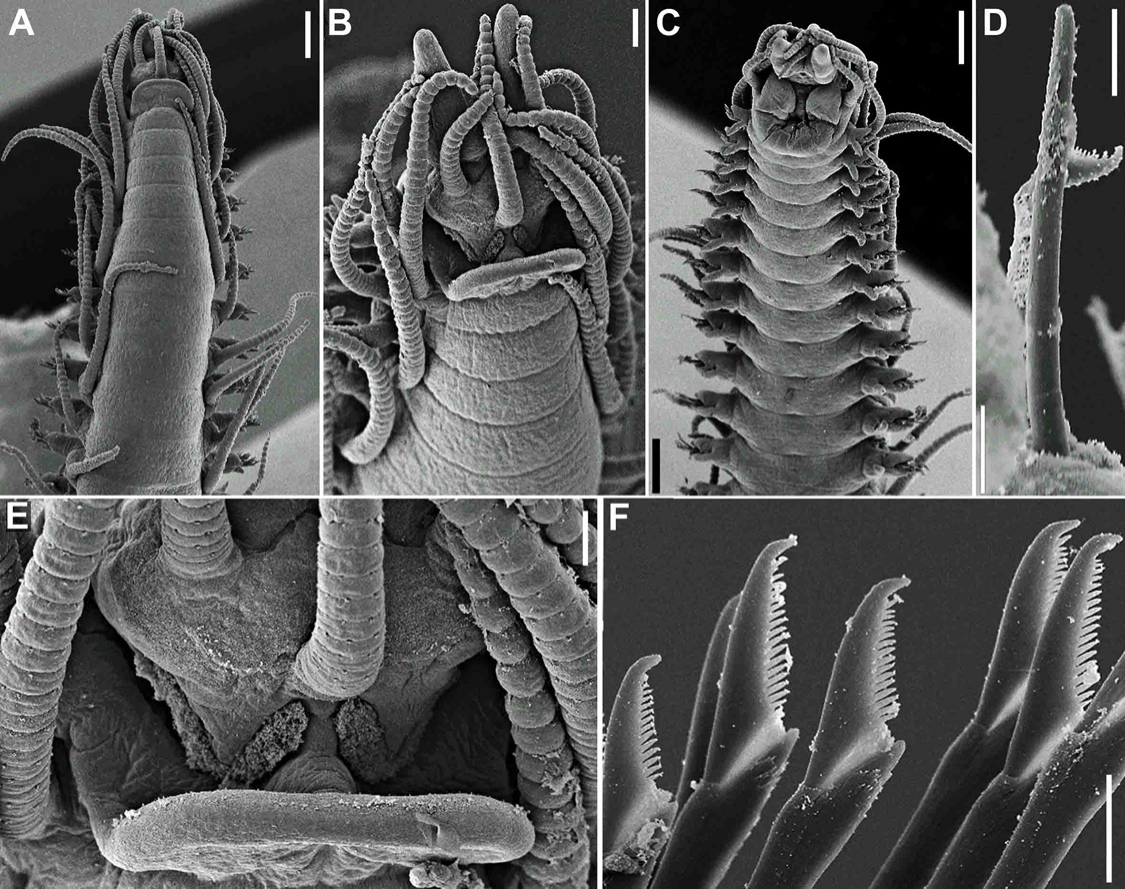
*Opisthosyllis brunnea*, SEM. (A) (B) anterior body, dorsal view; (C) anterior body, ventral view; (D) dorsal simple chaeta, midbody; (E) detail of prostomium, dorsal view; (F) chaetae, anterior body. Scale bars: A, C, 300 μm; B, 100 μm; D, F, 10 μm; E, 50 μm.

**Fig 21:**
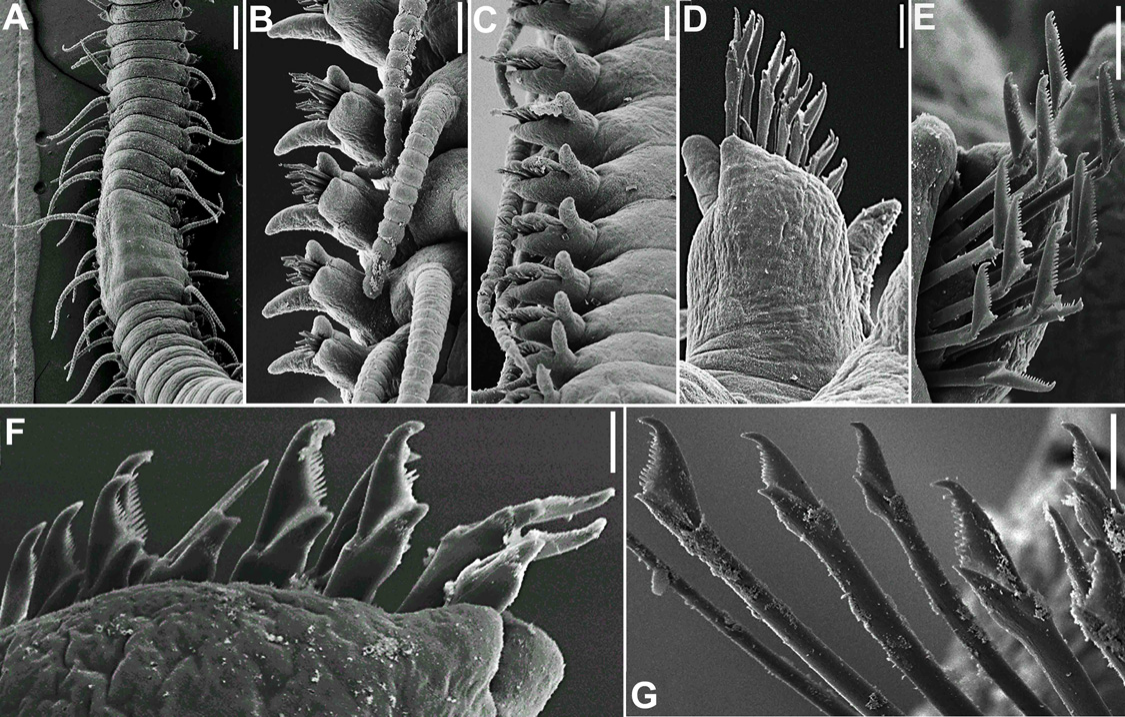
*Opisthosyllis brunnea*, SEM. (A) midbody segments, dorsal view; (B) (C) anterior parapodia, dorsal and ventral views, respectively; (D) anterior parapodium, dorso-lateral view; (E) (F) (G) chaetae, anterior,midbody and posterior body parapodia, respectively. Scale bars: A, 400 μm; B–C, 100 μm; D, 40 μm; E, 20 μm; F–G, 10 μm.

*Opisthosyllis brunnea* Langerhans, 1879: 541, Fig 7 [36]. Augener, 1918: 274, Fig 25 [37]. Day, 1967: 253, Fig 12.5 C–E [38]. Cantone, 1976: 229, Figs 2–3 [39]. Fauvel, 1953: 155, Fig 78 [40]. Hartmann-Schröder, 1979: 86; 1980: 48 [41]; 1981: 24 [42]; 1982: 58 [43]; 1991: 25, Fig 19 [44]. San Martín, 1984: 311, Figs 75–76 [26]; 1991: 230 [45]; 2003: 330, Fig 183 [2]. Núñez *et al*., 1993: 115, Figs 2H, 3F [46]. Capa *et al*., 2001: 106 [47].

Not *Opisthosyllis brunnea*. Imajima, 1966: 230, Fig 42 [48]. Lee & Rho, 1994: 135, Fig 2 [49].

*Opisthosyllis nuchalis* Verrill, 1900: 620 [50]. Monro, 1933: 254, Fig 7A–C [51].

*Opisthosyllis australis* Augener, 1913 (in part): 218, pl. 28, Fig 35 [52].

#### Material examined

Project ‘*BioPol-NE*’. State of Paraíba: João Pessoa, Praia de Cabo Branco (07^o^08'S 34^o^47'W), intertidal: 1 specimen (MZUSP 2147), coll. 09 February 2009; 8 specimens (MZUSP 2146), coll. 02 February 2010. Conde, Praia de Carapibus (07º17'S 34º48'W), intertidal: 9 specimens (MZUSP 2148), on rodholiths: 2 specimens (MZUSP 2149), coll. 10 February 2009; Praia do Coqueirinho (07º18’S 34º47’W), 2 specimens (MZUSP 2150), coll. 28 August 2011; Praia de Tambaba (07°21'S 34°47'W), 1 specimen (MZUSP 2151), coll. 30 August 2011. State of Pernambuco: Ilha de Itamaracá, recifes de Itamaracá, (07°43.944'S 34°49.200'W), 1 m: 1 specimen (MZUSP 2153), coll. 15 December 2012. Project ‘*Poly-Phytal’* State of Espírito Santo: Vitória, Ilha do Boi, Praia da Direita (20°18'S 40^o^17'W), rocky shore, intertidal: on *Arthrocardia gardnerii* Manza, 1937: 14 specimens (MZUSP 2199), coll. 21 July 2005; 14 specimens (MZUSP 2205), coll. 01 November 2005; 13 specimens (MZUSP 2201), coll. 01 March 2006; on *Centroceras clavulatum* (C. Agardh) Montagne, 1846: 5 specimens (MZUSP 2197), coll. 26 May 2005; 1 specimen (MZUSP 2145), coll. 01 November 2005; 1 specimen (MZUSP 2200), coll. 01 March 2006; on *Colpomenia sinuosa* (Mertens ex Roth) Derbès & Solier, 1851: 17 specimens (MZUSP 2206), coll. 01 November 2005; 9 specimens (MZUSP 2204), coll. 01 March 2006; on *Hypnea musciformis* (Wulfen) J. V. Lamouroux 1813: 13 specimens (MZUSP 2198), coll. 26 May 2005; 4 specimens (MZUSP 2195), coll. 21 July 2005; 2 specimens (MZUSP 2207), coll. 01 November 2005; 8 specimens (MZUSP 2202), coll. 01 March 2006; on *Sargassum* sp.: 1 specimen (MZUSP 2194), coll. 26 May 2005; 1 specimen (MZUSP 2203), coll. 01 March 2006.

#### Additional material examined

*Opisthosyllis brunnea*. Japan, Hokkaido, Rishiri Island (45°11.3'N 141°08.1'E), intertidal: 8 specimens (NSMT 3518–3525), coll. August 1963, coll. & det. M. Imajima. Venezuela, Falcón, Tiraya, Península de Paraguaná, 0.5 m: 1 specimen (MNCN 16.01/11025), coll. 10 January 2007, coll. & det. Díaz & Vanegas. Cuba, Archipiélago de los Canarreos, Isla de Pinos, Cayo Matías: 1 specimen (MNCN 16.01/750), coll. 18 May 1984, coll. & det. G. San Martín

#### Description

Large-sized body, longest specimen analysed 23.8 mm long, 1.1 mm wide, with 81 chaetigers. Brownish green body, especially anteriorly. Palps triangular, distally pointed (Fig 20A–20C), totally free from each other. Prostomium subpentagonal, as long as palps (Fig 20A and 20B), with two pairs of eyes in open trapezoidal arrangement, anterior eyespots absent; median antenna inserted between posterior pair of eyes, reaching beyond tip of palps, with 23–26 articles; lateral antennae shorter, inserted anteriorly to eyes, shorter than median antenna (Fig 20A and 20B and 20E), with 13–19 articles each. Ciliated nuchal organs dorsally between prostomium and peristomium, distinctly curved, reaching base of median antenna, under occipital flap, and extending laterally around prostomium/peristomium border (Fig 20A and 20B and 20E). Peristomium shorter than anterior chaetigers, with rounded, nearly semicircular occipital flap with fringe of cilia on anterior border, covering posterior part of prostomium (Fig 20A and 20B and 20E); dorsal peristomial cirri as long as median antennae or slightly longer, with 26–30 articles each; ventral peristomial cirri shorter, with 11–18 articles each. Dorsal cirri of chaetiger 1 longer than dorsal peristomial cirri, with 36–40 articles each; dorsal cirri of chaetigers 2 and 5 shorter, of similar size, with 17–21 articles each; dorsal cirri of chaetigers 3 and 4 with 29–31 and 33–36 articles each, respectively; following dorsal cirri alternating long, with 32–38 articles each, longer than body width at corresponding segment, and short, with 23–29 articles each, shorter than body width at corresponding segment (Figs 20A; 21A); dorsal cirri with broader proximal articles and tapered distal articles (Figs 20A and 20B; 21A and 21B). Antennae, peristomial and dorsal cirri throughout with short cirrophores. Ventral cirri digitiform, inserted at beginning of middle third of parapodial lobes and reaching their tips (Figs 20C; 21C). Parapodial lobes distally bilobed, pre-chaetal lobe slightly longer (Fig 21B–21D). Anterior parapodia with 10–16 falcigers each; midbody parapodia with 9–13, posterior parapodia with 5–11 falcigers each. Shafts of falcigers slightly spinulated subdistally (Figs 20F; 21F and 21G); tips of shafts straight on anterior body (Fig 20F) and sigmoid on mid- and posterior body, especially on ventralmost chaetae of posterior body chaetigers (Fig 21F and 21G); blades of falcigers spinulated, unidentate to subbidentate, the latter with conspicuous subdistal spine (Figs 20F; 21E–21G); falciger blades with slight gradation in length (Fig 21D–21G), blades 38–25 μm long on anterior parapodia, 34–24 μm and 30–20 μm long, on mid- and posterior body parapodia, respectively. Dorsal simple chaetae present from midbody, thinner than shafts of falcigers, distally truncate and slightly rounded, with minute subdistal spines (Fig 20D); ventral simple chaetae only present on posteriormost parapodia, sigmoid, unidentate, subdistally spinulated, about as thick as shafts of falcigers. Anterior parapodia with up to 4 aciculae each, straight, with hollow concavity; number of aciculae decreasing posteriorwards to single acicula per parapodium on posteriormost chaetigers. Pygidium semicircular, with two elongated anal cirri, as long as posterior dorsal cirri. Pharynx through 9–11 segments; large, oblong pharyngeal tooth near posterior margin of pharynx, visible through body wall in life; proventricle extending for 11–12 chaetigers, with 54–64 rows of muscle-cells.

#### Remarks

Brazilian specimens of *O*. *brunnea* are similar to those from the Caribbean (Venezuela and Cuba), Iberian Peninsula [2], Australia [53] and Japan, although with slight differences among specimens from different localities. Brazilian and Australian specimens have up to four aciculae per parapodium anteriorly, while Caribbean and Iberian specimens have only two. Brazilian, Caribbean and Iberian specimens have falciger blades with subdistal tooth as a spine (subbidentate), and ca. 60 rows of muscle cells in the proventricle, while Australian specimens have bidentate blades of anterior body falcigers, with distal tooth larger than subdistal one, and ca. 30 rows of muscle cells in the proventricle. These discrepancies indicate that *O*. *brunnea*, as currently recognized, may be a complex of sibling species.

#### Type locality

Madeira Island, Portugal (Atlantic Ocean).

#### Distribution

Pacific Ocean: Japan, Australia (Queensland and New South Wales). Indian Ocean: Australia (Western Australia), Mozambique, South Africa. Atlantic Ocean: Mediterranean Sea (Spain: Baleares and Chafarinas Islands), Portugal (Madeira Island), Brazil (Paraíba to São Paulo). First record from off Espírito Santo and northeastern Brazilian coast.

*Opisthosyllis viridis* Langerhans, 1879

Figs 22–24

**Fig 22:**
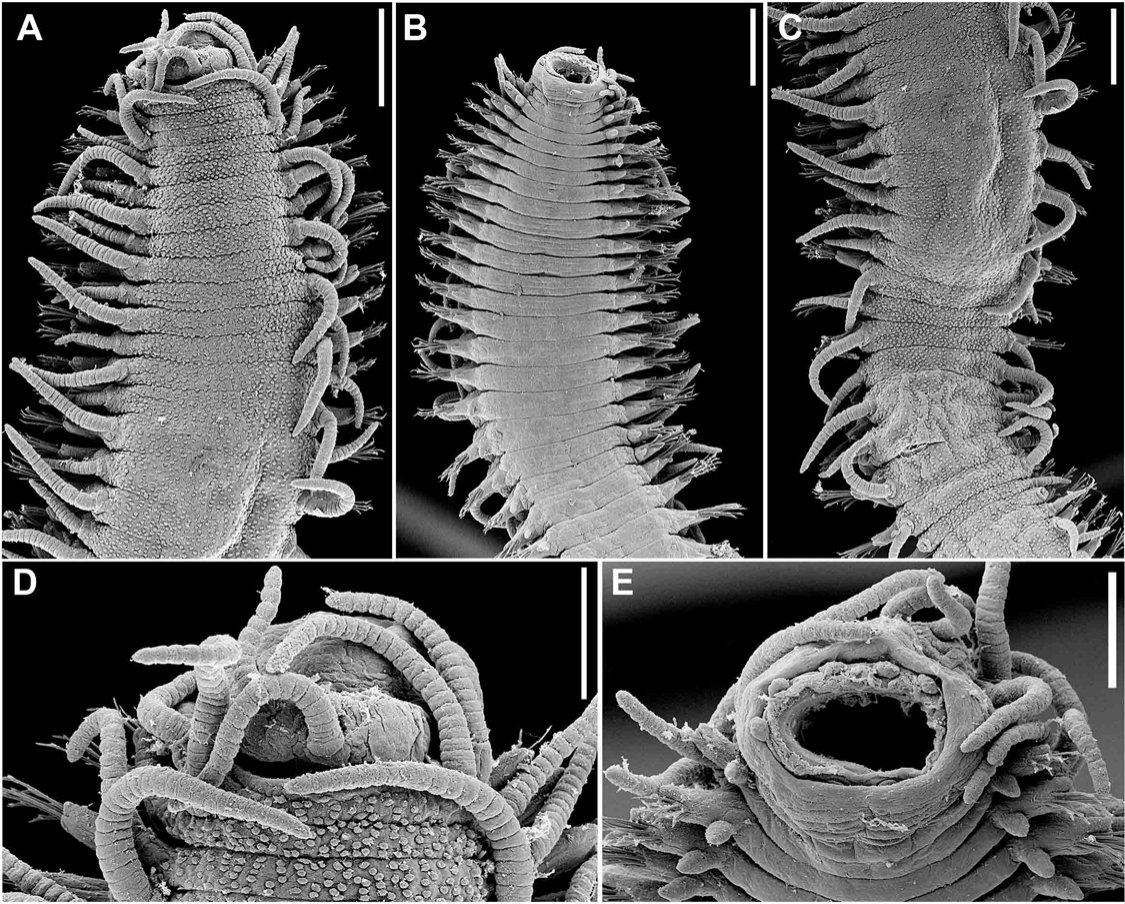
*Opisthosyllis viridis*, SEM. (A) anterior body, dorsal view; (B) anterior body, ventral view; (C) midbody, dorsal view; (D) anterior end, dorsal view; (E) anterior end, fronto-ventral view. Scale bars: A–C, 200 μm; D–E, 100 μm.

**Fig 23:**
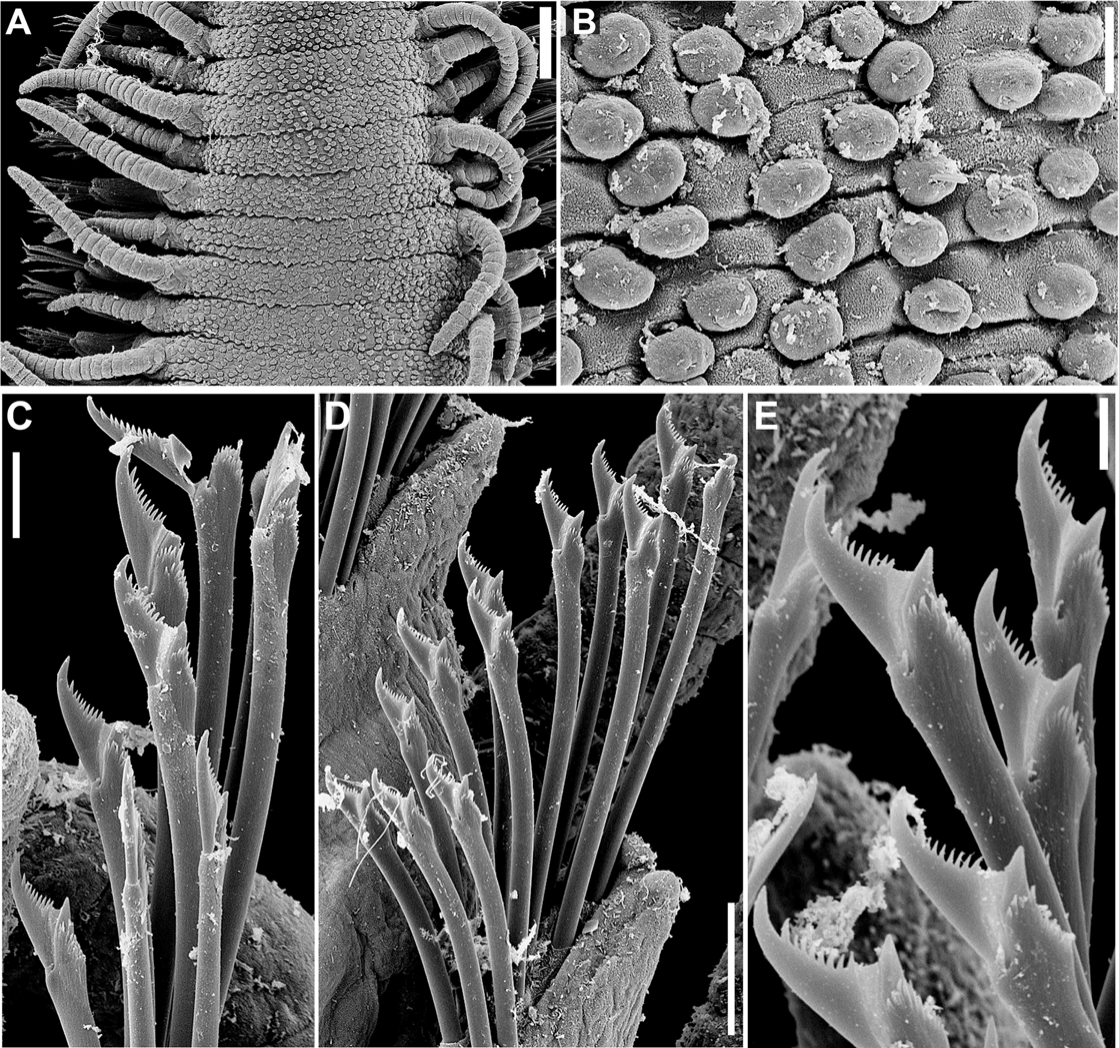
*Opisthosyllis viridis*, SEM. (A) midbody, dorsal view; (B) detail of papillae, midbody, dorsal view; (C) (D) (E) chaetae, anterior, midbody and posterior parapodia, respectively. Scale bars: A, 100 μm; B, 20 μm; C–D, 10 μm; E, 4 μm.

**Fig 24:**
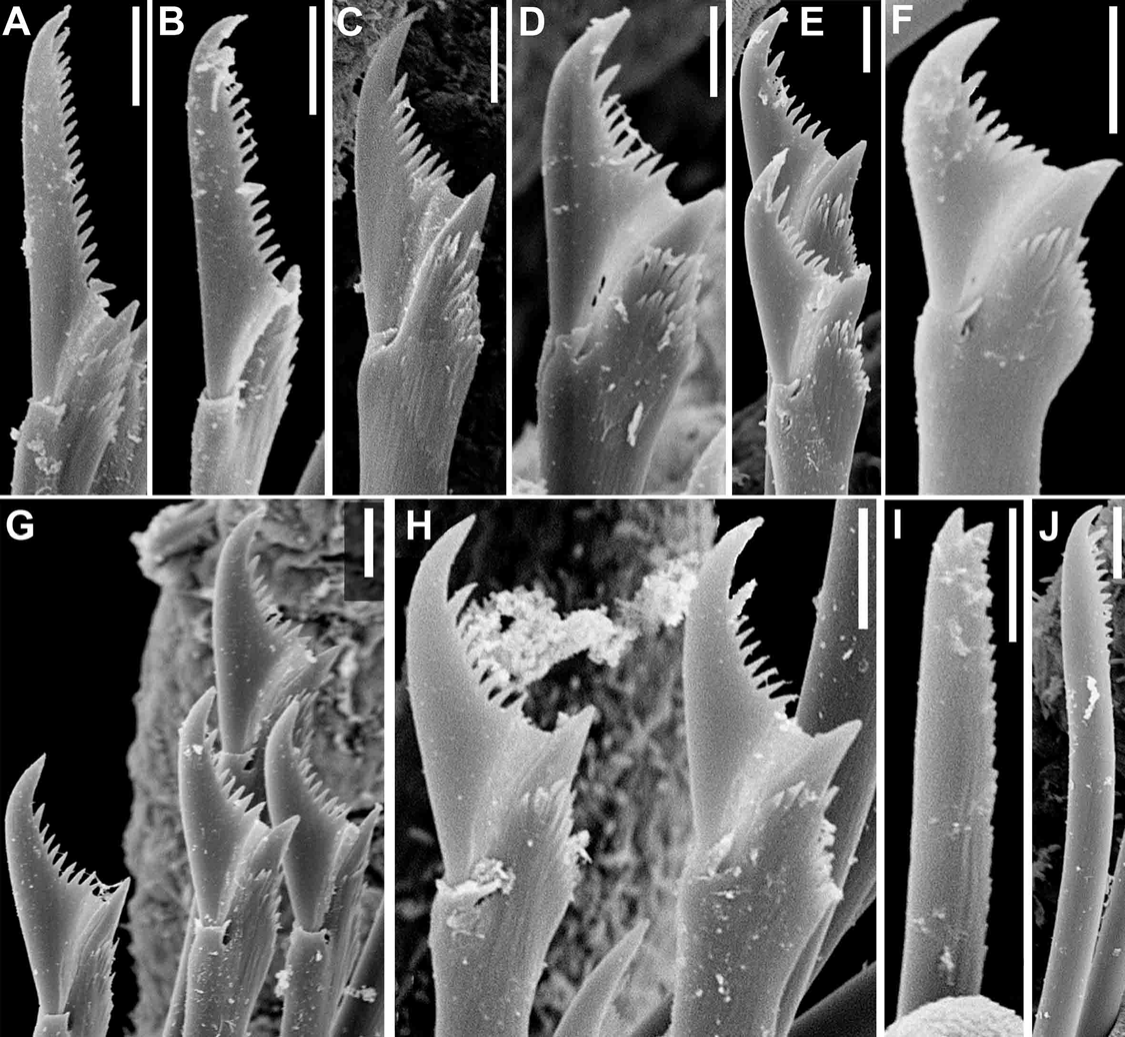
*Opisthosyllis viridis*, SEM. (A) dorsalmost falciger, anterior parapodium; (B) intermediate falciger, anterior parapodium; (C) ventralmost falciger, anterior parapodium; (D) dorsalmost falciger, midbody parapodium; (E) intermediate falcigers, midbody parapodium; (F) ventralmost falciger, midbody parapodium; (G) dorsalmost falcigers, posterior parapodium; (H) ventralmost falcigers, posterior parapodium; (I) dorsal simple chaeta, posterior parapodium; (J) ventral simple chaeta, posterior parapodium. Scale bars: A–J, 4 μm.

*Opisthosyllis viridis* Langerhans, 1879: 543, pl. 31, Fig 9 [36]. Monro, 1939: 390, Fig 301 [54]. Imajima, 1966: 224, Fig 39 [48]. López & San Martín, 1994: 130, Fig 1 [55]. Lee & Rho, 1994: 135, Fig 3 [49].

*Opisthosyllis australis* Augener, 1913 (in part): 218, pl. 3, Fig 35, Fig 28 a–d [52]. Hartmann-Schröder, 1979: 85 [41]; 1980: 47 [56]; 1981: 24 [42]; 1991: 26[44].

*Opisthosyllis papillosa* Hartmann-Schröder, 1960: 87, Figs 59–62 [57]; 1965: 108, Figs 33–35 [58]; 1980: 48 [56]; 1981: 24 [42]; 1991: 26 [44].

#### Material examined

Project ‘*BioPol-NE*’. State of Pernambuco: Ilha de Itamaracá, Ponta do Jaguaribe (07°44'S 34°49'W), intertidal: 2 specimens (MZUSP 2154), coll. 11 December 2012. State of Espírito Santo: Vitória, Ilha do Boi, Praia da Direita (20°18'S 40°17'W), rocky shore, intertidal, on *Arthrocardia gardnerii*: 1 specimen (MZUSP 2192), coll. 26 May 2005; 2 specimens (MZUSP 2158), coll. 21 July 2005; on *Centroceras clavulatum*: 7 specimens (MZUSP 2193), coll. 26 May 2005; 1 specimen (MZUSP 2159), coll. 01 November 2005; 4 specimens (MZUSP 2189), coll. 01 March 2006; on *Hypnea musciformis*: 14 specimens (MZUSP 2191), coll. 26 May 2005; 1 specimen (MZUSP 2190), coll. 01 March 2006; on *Sargassum* sp.: 1 specimen, coll. 26 May 2005; 1 specimen, coll. 01 March 2006. Project ‘*BIOTA’*: State of São Paulo: Ubatuba, Praia de Picinguaba (23°22'31"S 44°50'21"W), intertidal: 1 specimen (MZUSP 2160), coll. 08 June 2001. São Sebastião, Praia de Toque-Toque Grande (23°50'12''S 45°30'40''W), intertidal: 1 specimen (MZUSP 2161), coll. 10 April 2001; Praia da Baleia (23°46.800'S 45°39.850'W), intertidal: 4 specimens (MZUSP 2155), coll. 08 April 2001; 6 specimens (MZUSP 2156), coll. 12 December 2001; 1 specimen (MZUSP 2157), coll. 17 October 2005. Project ‘*BioPol-SP’*: Guarujá, Ilha das Palmas (24°00'34''S 46°19'25''W), intertidal: 1 specimen (MZUSP 2162), coll. 05 October 2005.

#### Additional material examined

*Opisthosyllis leslieharrisae* Aguado, San Martín & Nygren, 2005. USA, California, Isla de Santa Catalina (33º26.7'N 118º29.1'W), 1–4 m: holotype (MNCN 16.01/10264) and 1 paratype (MNCN 16.01/10266), coll. 15 January 2001, A. Nygren and J. Toth, det. M.T. Aguado, G. San Martín & A. Nygren.

*Opisthosyllis viridis*. Japan, Kochi, Usa (33°26.2'N 133°26.9'E), intertidal: 6 specimens (NSMT 3506–3511) coll. May 1965, coll. & det. M. Imajima; Hiroshima, Mukaishima (34°21.7'N 133°13.2'E), intertidal: 1 specimen (NSMT 3516), coll. June 1964, coll. & det. M. Imajima; Ishikawa, Noto Penin (37°18.3'N 137°14.4'E), intertidal, 4 specimens (NSMT 3498–3501, 3517), coll. July 1964, coll. & det. M. Imajima.

#### Description

Relatively small-sized body, longest specimen analysed 8 mm long, 0.6 mm wide, with 74 chaetigers. Body without colour patterns; dorsal surface of peristomium and chaetigers throughout covered with numerous rounded, small papillae, arranged in irregular, transverse rows (Figs 22A, 22C and 22D; 23A and 23B). Palps roughly triangular, distally rounded, fused at bases. Prostomium ovate, shorter than palps, with two pairs of eyes in open trapezoidal arrangement, anterior eyespots absent; median antenna inserted between posterior pair of eyes, near posterior border of prostomium, reaching beyond tip of palps, with 17–21 articles; lateral antennae shorter, inserted on anterior margin of prostomium, with 9–14 articles each (Fig 22A and 22D). Nuchal organs not visible. Peristomium shorter than anterior chaetigers, occipital flap absent; dorsal peristomial cirri as long as median antenna or slightly longer, with 19–25 articles each; ventral peristomial cirri shorter, with 11–15 articles each. Dorsal cirri of chaetiger 1 longer than dorsal peristomial cirri, with 23–30 articles each; dorsal cirri of chaetigers 2 and 5 of similar size, shorter, with ca. 11 articles each; dorsal cirri of chaetigers 3 and 4 with 12–18 articles each; following dorsal cirri alternating long, with ca. 18 articles each, as long as ¾ body width at corresponding segment, and short, with ca. 9 articles each, about ½ body width at corresponding segment; posterior dorsal cirri shorter, long and short cirri with 6–9 and 2–6 articles each, respectively, both shorter than ½ body width at corresponding segment (Figs 22C and 23A). Antennae, peristomial and dorsal cirri with short cirrophores. Ventral cirri ovate on anterior body (Fig 22B and 22E), more digitiform and elongated posteriorwards, shorter than parapodial lobes, inserted at their bases. Parapodial lobes conical, slightly bilobed (Figs 22A–22E; 23A and 23D). Anterior parapodia with 14–17 falcigers each; midbody parapodia with 8–11, posterior parapodia with 4–6 falcigers each. Shafts of falcigers slightly spinulated subdistally (Figs 23C–23E; 24A–24H); shafts of falcigers on anterior body with straight tips, slightly inflated subdistally (Figs 23C; 24A–24C), shafts with more sigmoid tips posteriorwards (Figs 23D–23E; 24D–24H); blades of falcigers with minute spines, subbidentate, with conspicuous subdistal spine (Figs 23C–23E, 24A–24H); blades of falcigers with dorso-ventral gradation in length, 29–10 μm long, 20–15 μm long and 12–10 μm long on anterior, mid- and posterior body parapodia, respectively. Dorsal simple chaetae present from midbody, as thick as shafts of falcigers, distally bifid, with minute subdistal spines (Fig 24I); ventral simple chaetae only present on posteriormost parapodia, thinner than shafts of falcigers, sigmoid, bidentate, slightly spinulated subdistally (Fig 24J). Anterior parapodia with up to 2 aciculae each, with slightly inflated tips; number of aciculae decreasing posteriorwards to single acicula per parapodium on posteriormost chaetigers. Pygidium semicircular, with pair of elongate anal cirri, longer than posterior dorsal cirri, with 11–13 articles each. Pharynx through 7–9 segments, surrounded by crown of 10–12 soft papillae and ring of cilia (Fig 22E); pharyngeal tooth near posterior margin of pharynx; proventricle extending for 6–7 chaetigers, with ca. 52 rows of muscle cells.

#### Remarks

*Opisthosyllis leslieharrisae* and *O*. *convexa* Lee & Rho, 1994 also have the dorsal surface covered by numerous papillae. Brazilian specimens of *O*. *viridis* can be differentiated from *O*. *leslieharrisae* by having rounded papillae, shorter dorsal cirri, distinctly shorter spines on the edge of the falciger blades, and pharyngeal tooth located more posteriorly in the pharynx, close to the proventricle, while *O*. *leslieharrisae* has triangular papillae on dorsum and the pharyngeal tooth located at 3/4 pharyngeal length.

*Opisthosyllis convexa* has longer appendages than the Brazilian specimens of *O*. *viridis*. Furthermore, in *O*. *convexa*, the subdistal tooth of falcigers is more separated from the distal one than in *O*. *viridis*, and the pharynx occupies 5–6 chaetigers, while the proventricle extends for 10–11 chaetigers [49].

*Opisthosyllis papillosa* Hartmann-Schröder, 1960 and *O*. *australis* Augener, 1913, were recently recognized as junior-synonymes of *O*. *viridis* [53]. The authors [53] noticed that *O*. *viridis*, as currently considered, has high intraspecific variability, suggesting that this could be another case of a complex of sibling species. Brazilian specimens of *O*. *viridis* also present some differences from specimens from others localities, with shorter appendages than the described for Australian [53] and Japanese material; bifid dorsal simple chaetae and bidentate ventral simple chaetae; and proventricle with ca. 52 rows of muscle cells. Also different from the Brazilian material, the specimens analysed from Japan have an occipital flap. On the other hand, Australian specimens have bidentate dorsal simple chaetae and ventral simple chaetae with acute tip (Fig 27G in [53]); and proventricle with ca. 32 rows of muscle cells. Korean specimens of *O*. *viridis* have dorsal simple chaetae similar to those of the Brazilian specimens (bifid) and ventral simple chaetae similar to those of the Australian specimens (with acute tip) [49]. Papillae are absent on the prostomium and palps of the Brazilian and the Australian specimens (Fig 27 in [53]), but present in material from Cape Verde [55]. Because of this great variability observed in the material of *O*. *viridis* from different localities, we agree with [53] in that a molecular approach is needed to clarify the diversity of species currently under this name.

#### Type locality

Madeira Island, Portugal (Atlantic Ocean).

#### Distribution

Pacific Ocean: South Korea, Japan, Australia (Queensland and New South Wales). Indian Ocean: Australia (Western Australia). Atlantic Ocean: Portugal (Madeira Island) and Brazil (Pernambuco, Espírito Santo and São Paulo). First record from off the northeastern Brazilian coast.

Genus *Trypanosyllis* Claparède, 1864

Type species: *Syllis zebra* Grube, 1860, designated by Claparède (1864).

#### Diagnosis

Medium to large sized syllines, up to 13 cm long, with flattened, ribbon-like body. Palps ovate, free from each other. Prostomium with 3 antennae, 2 pairs of eyes, sometimes with pair of anterior eyespots. Antennae, peristomial and dorsal cirri throughout articulated. Peristomium usually dorsally reduced, with two pairs of peristomial cirri. Compound chaetae as falcigers only, sometimes secondarily simple due to fusion of shafts and blades; dorsal and/or ventral simple chaetae sometimes present on posteriormost parapodia. Pharynx with anterior trepan; central, larger tooth sometimes also present. Reproduction by means of *Tetraglene* stolons [32].

#### Remarks

Three species of *Trypanosyllis* were recorded from Brazil: *T*. *aurantiacus* Nogueira & Fukuda, 2008, originally described from the state of São Paulo; *T*. *parvidentata* Perkins, 1981 found in the Guanabara Bay, state of Rio de Janeiro [59]; and *T*. *zebra*, recorded from the states of Pernambuco and Bahia (northeastern Brazilian coast) [22], and São Paulo [13,27,35,60] and Rio de Janeiro [12,61] (southeastern Brazilian coast).

*Trypanosyllis zebra* (Grube, 1860)

*Syllis zebra* Grube, 1860: 86, pl. 3, Fig 7 [62].

*Trypanosyllis zebra*. Langerhans, 1879: 556 [36]. Haswell, 1920: 101 [63]. Fauvel, 1923: 269, Figs 101A–E [64]. Day, 1967: 256, Figs 12.6. A–B [38]. San Martín, 2003: 311, Figs 171–173 [2]. Nogueira & Fukuda, 2008: 2–7, Fig 1 [13]. San Martín *et al*., 2008: 43–48, Figs 31F, 32–36 [53].

*Trypanosyllis krohnii* Claparède, 1864: 558, pl. 7, Fig 2 [65].

*Syllis taeniaformis* Haswell, 1885: 741, pl. 1, Figs 4–5 [66].

*Trypanosyllis taeniaformis* Augener, 1913: 230 [52]. Westheide, 1974: 39, Figs 16A–D [67]. Day & Hutchings, 1979: 105 [68].

*Trypanosyllis* (*Trypanedenta*) *taeniaformis* Imajima & Hartman, 1964: 127, Figs 30 H–K [69]. Imajima, 1966: 239, Figs 45 A–I [48]. Hartmann-Schröder, 1989: 18 [70].

*Trypanosyllis richardi* Gravier, 1900: 168, pl. 9, Figs 12–13 [71].

*Trypanosyllis vittigera* Ehlers, 1887: 151, pl. 40, Figs 1–3 [72]. Uebelacker, 1984: 30–88, Figs 30–81, 82A–H [73].

*Parautolytus luzonensis* Pillai, 1965: 123, Figs 5E–I, 6A–C [74].

#### Material examined

Project ‘*BioPol-NE*’. State of Paraíba: Mataraca, Barra de Camaratuba (06º36'S 34º57'W), intertidal: 4 specimens (MZUSP 2174), coll. 12 August 2010. Baía da Traição, Praia do Farol (06º41'S 34º55'W), intertidal: 3 specimens (MZUSP 2173), coll. 09 August 2010. Rio Tinto, Barra de Mamanguape (06º45'S 34º55'W), intertidal: 4 specimens (MZUSP 2175), coll. 11 August 2010. Cabedelo, Píer de Cabedelo (06º58'S 34º50'W), intertidal: 1 specimen (MZUSP 2179), coll. 12 February 2009. João Pessoa, Praia de Cabo Branco (07^o^08'S 34^o^47'W), intertidal: 1 specimen (MZUSP 2165), coll. 09 February 2009; 1 specimen (MZUSP 2181), coll. 02 February 2010. Conde, Praia de Jacumã (07º14'S 34º47'W), intertidal: 1 specimen (MZUSP 2166), coll. 29 January 2010; Praia de Tabatinga (07º19'S 34º47'W), intertidal: 3 specimens (MZUSP 2186), coll. 1 September 2011; on *Amphimedon viridis*: 1 specimen (MZUSP 2185), coll. 17 September 2012; Praia de Tambaba (07°21'S 34°47'W), 1 specimen (MZUSP 2163), coll. 30 August 2011. State of Pernambuco: Goiana, Pontas de Pedra (07°37'S 34°48'W), 4 specimens (MZUSP 2170), coll. 13 December 2012. Ilha de Itamaracá, Ponta do Jaguaribe (07°44'S 34°49'W), intertidal: 1 specimen (MZUSP 2182), coll. 11 December 2012. State of Espírito Santo: Vitória, Ilha do Boi, Praia da Direita (20^°^18'S 40^o^17'W), rocky shore, intertidal, on *Arthrocardia gardnerii*: 12 specimens (MZUSP 2172), coll. 21 July 2005; on *Hypnea musciformis*: 1 specimen (MZUSP 2180), coll. 26 May 2005. Project ‘*BIOTA*’ State of São Paulo: Ubatuba, Praia de Picinguaba (23°22'31"S 44°50'21"W), intertidal: 57 specimens (MZUSP 2183), coll. 08 June 2001; 39 specimens (MZUSP 2169), coll. 18 October 2001; 2 specimens (MZUSP 2188), coll. 17 October 2001. São Sebastião, Praia da Baleia (23°46.800'S 45°39.850'W), intertidal: 20 specimens (MZUSP 2184), coll. 23 July 2005; 1 specimen (MZUSP 2187), coll. 17 October 2005; Praia do Araçá (23°48'54"S 45°24'24"W), intertidal: 1 specimen (MZUSP 2164), coll. 20 July 2005. Project *‘BioPol-SP’* Guarujá, Praia de Pernambuco (23°58'20"S 46°11'02"W), intertidal: 22 specimens (MZUSP 2168), coll. 22 June 2005; 8 specimens (MZUSP 2167), coll. 04 October 2005; Ilha das Palmas, (24°00'34"S 46°19'25"W), intertidal: 1 specimen (MZUSP 2176), coll. 06 March 2004. São Vicente, Ilha Porchat (23°58'39"S 46°22'08"W), intertidal: 1 specimen (MZUSP 2177), coll. 18 November 2002.

#### Additional material examined

State of Rio de Janeiro: Búzios, Praia Azeda, intertidal: 1 specimen (MZUSP 2171), coll. 08 April 2010.

#### Diagnosis

*Trypanosyllis* with two transverse, red bands on dorsum of each anterior segment; dorsal cirri relatively elongate, frequently red, as long as or slightly longer than body width, at least on anterior body; compound chaetae bidentate, with teeth about same size and rounded space inbetween, with conspicuous dorso-ventral gradation in length of blades.

#### Remarks

The specimens analysed herein agree with the description of Brazilian material provided by Nogueira & Fukuda (2008).

#### Type locality

France (Mediterranean Sea).

#### Distribution

Atlantic Ocean: Mediterranean Sea, Africa, Brazil (states of Paraíba, Pernambuco, Espírito Santo, Rio de Janeiro and São Paulo). This is the first record for this genus to the states of Paraíba and Espírito Santo.
